# Supplementary material for: Machine learning can identify newly diagnosed patients with CLL at high risk of infection
Source: Nat Commun. 2020 Jan 17;11:363. doi: 10.1038/s41467-019-14225-8 (PMC6969150; doi:10.1038/s41467-019-14225-8)
Supplement: Supplementary file 1 — Supplementary Information [file 41467_2019_14225_MOESM1_ESM.pdf]

Supplementary Information

'Machine Learning can Identify Newly Diagnosed Patients with CLL at High Risk of Infection'

Agius et. al

Supplementary Figures

- Supplementary Figure 1. Consort Diagram
- Supplementary Figure 2. Distribution of time-point zero for CLL-TIM's predictions on external cohort
- Supplementary Figure 3. Infection date modelling features
- Supplementary Figure 4. Ensemble Scoring and Ranking
- Supplementary Figure 5. CLL-TIM feature redundancy analysis
- Supplementary Figure 6. Event free survival and cumulative incidence plots for CLL-TIM on internal test cohort
- Supplementary Figure 7. Ensemble benchmark models
- Supplementary Figure 8. Robustness Simulations on internal test cohort
- Supplementary Figure 9. Missing data conditions in external cohort compared to internal cohort
- Supplementary Figure 10. Cumulative incidence for the Danish internal test cohort and the German CLL7 external cohort
- Supplementary Figure 11. Internal test cohort simulations of missing data conditions of CLL7 external cohort
- Supplementary Figure 12. Distribution of CLL-TIM's probabilistic output on addition of patient data
- Supplementary Figure 13. Statistical testing for modelling infection and CLL treatment separately and jointly
- Supplementary Figure 14. Detection of Infection prior to Treatment with CLL-TIM and CLL-IPI
- Supplementary Figure 15. UMAP clustering for personalized high-risk factors in CLL-TIM
- Supplementary Figure 16. Variable-to-risk mapping learnt by CLL-TIM for Rare Pathology
- Supplementary Figure 17. Genetic algorithm ensemble generation

Supplementary Tables

- Supplementary Table 1. Modeling of Lab Test Values and Dates
- Supplementary Table 2. Variables and Feature Encodings Summary
- Supplementary Table 3. Base-Learner hyper parameters and feature selection methods
- Supplementary Table 4. Risk of infection or treatment and event-free survival on Internal Test Cohort.
- Supplementary Table 5. Benchmark models description and motivations
- Supplementary Table 6. 2-Year Composite Outcome for BENCH-I
- Supplementary Table 7. Significance Testing for Model Comparison on 2-Year Composite Outcome for BENCH-I
- Supplementary Table 8. Significance Testing for Model Comparison on 2-Year Composite Outcome for BENCH-I
- Supplementary Table 9. 5-Year Composite Outcome for BENCH-I
- Supplementary Table 10. Significance Testing for Model Comparison on 5-Year Composite Outcome for BENCH-I
- Supplementary Table 11. Significance Testing for Model Comparison on 5-Year Composite Outcome for BENCH-I
- Supplementary Table 12. Risk of infection or treatment and event-free survival on External Cohort
- Supplementary Table 13. 2-Year Composite Outcome for BENCH-E
- Supplementary Table 14. Significance Testing for Model Comparison on 2-Year Composite Outcome for BENCH-E
- Supplementary Table 15. Significance Testing for Model Comparison on 2-Year Composite Outcome for BENCH-E
- Supplementary Table 16. 5-Year Composite Outcome for BENCH-E. Predictions
- Supplementary Table 17. Significance Testing for Model Comparison on 5-Year Composite Outcome for BENCH-E
- Supplementary Table 18. Significance Testing for Model Comparison on 5-Year Composite Outcome for BENCH-E
- Supplementary Table 19. 2-Year Treatment Outcome for BENCH-I.
- Supplementary Table 20. Significance Testing for Model Comparison on 2-Year Treatment Outcome for BENCH-I.
- Supplementary Table 21. Significance Testing for Model Comparison on 2-Year Treatment Outcome for BENCH-I.
- Supplementary Table 22. 2-Year Treatment Outcome for BENCH-E
- Supplementary Table 23. Significance Testing for Model Comparison on 2-Year Treatment Outcome for BENCH-E
- Supplementary Table 24. Significance Testing for Model Comparison on 2-Year Treatment Outcome for BENCH-E
- Supplementary Table 25. Baseline Variables used in this work and expansion using One-Hot-Encoding
- Supplementary Table 26. Ensemble Ranking Score

Supplementary Discussion

- Role of Immunoglobulins and Bag-of-Words Features

Supplementary Methods

- Feature Generation

Supplementary References

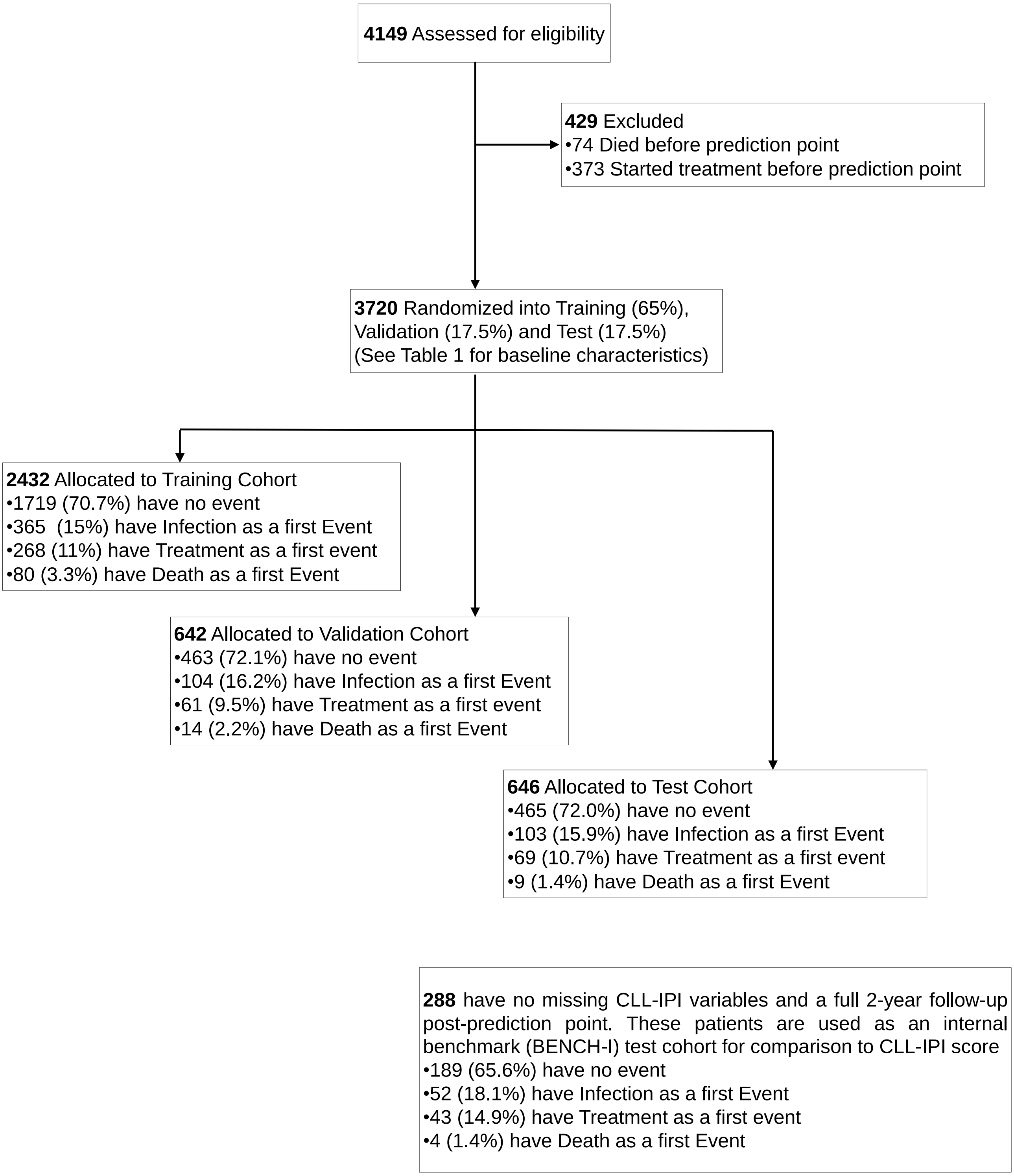

Supplementary Figure 1 | Consort Diagram. 4149 patients diagnosed with CLL in Denmark between January 2004 and June 2017 were eligible for this work. Prediction point refers to 3-months post-diagnosis. All available patient data up until prediction point is used for modeling the 2-year risk composite outcome of infection and/or treatment post prediction point. Train, Validation and Test Cohort randomization was performed using stratified sampling to control the ratio of composite events and patients with no missing CLL-IPI variables across the 3 cohorts. The allocation of ‘no event’, ‘Infection as a first Event’, ’Treatment as a first Event’ and ‘Death as a first Event’, were all restricted to events within the 2-year predictive window.

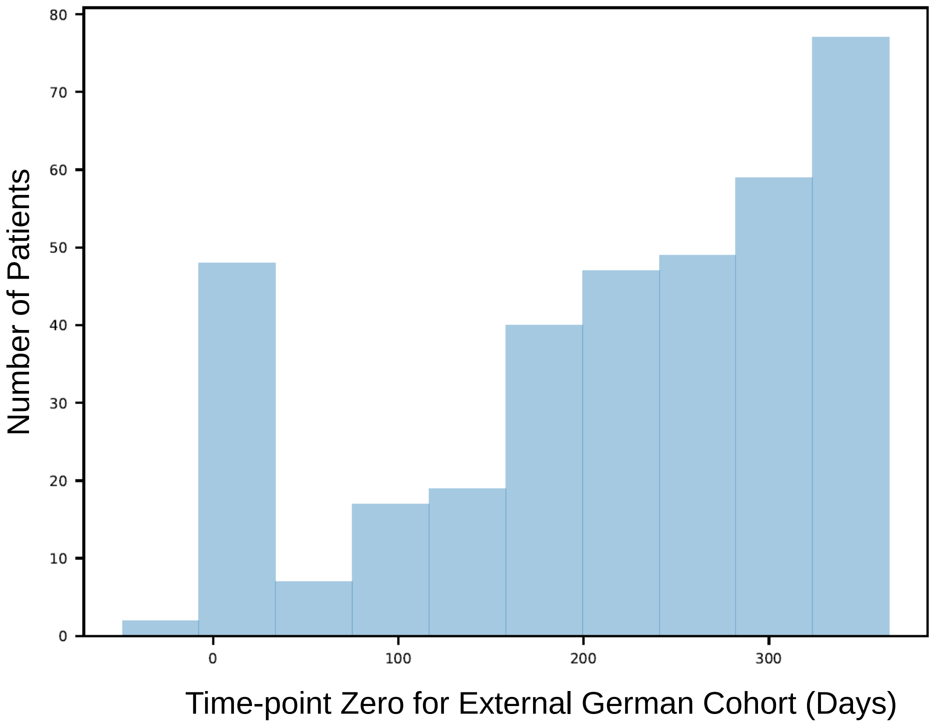

Supplementary Figure 2. **Distribution of time-point zero for CLL-TIM's predictions on external cohort.** Results are for (*n*=365) patients with baseline characteristics summarized in Table 1. To allow for tests ordered at time of diagnosis to be available, we trained CLL-TIM on patient data with time-point zero varied between zero to three months post diagnosis. However, there is no restriction for patients to be within the first three months of diagnosis for CLL-TIM's prediction. For example in the German cohort, the maximum time-point 0 for patients was extended up until one-year post-diagnosis. Given that the German cohort has very limited data prior do CLL-diagnosis (Supplementary Fig. 9), this allowed CLL-TIM to make use of any infection or laboratory data taken post CLL diagnosis for its predication.

a

Infection Time Distribution Modelling Features	Aim & Description
No. of Previous Infections	Number of Previous Infections
Mean No. of Days Between Infections	Density of Previous Infections
Standard deviation of Days Between Infections	Variability in Density of Previous Infections
Max No. of Days Between Infections	Density of Previous Infections
Min No. of Days Between Infections	Density of Previous Infections
Last Infection Date	Most Recent Infection
Earliest Infection Date	Earliest Infection
Infection Date LinearFit_coefa ($y=ax+b$)	Density & Recentness See (a) and (b)
Infection Date LinearFit_coefb ($y=ax+b$)	Density & Recentness See (a) and (b)
Infection Date QuadraticFit_coefa ($y=ax^2+bx+c$)	Density & Recentness See (a) and (b)
Infection Date QuadraticFit_coefb ($y=ax^2+bx+c$)	Density & Recentness See (a) and (b)
Infection Date QuadraticFit_coefc ($y=ax^2+bx+c$)	Density & Recentness See (a) and (b)
Infection Dates Mean	Recentness of Infections
Infection Dates Standard Deviation	Density of Previous Infections
Infection Date Kurtosis	Density and Recentness of Previous Infections
Infection Dates Skewness	Density and Recentness of Previous Infections

b

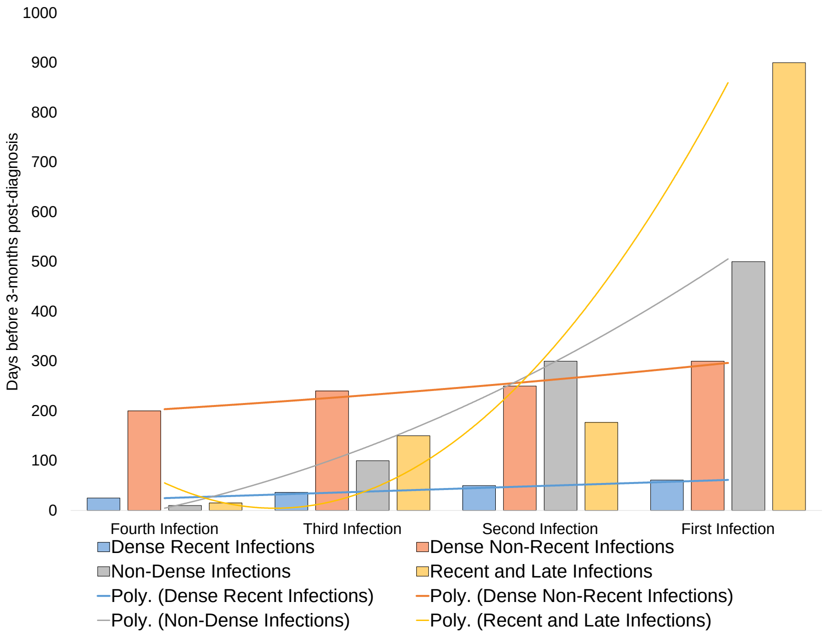

c

Patient	Fourth Infection (Days Before Prediction Point of 3-months Post Diagnosis)	Third Infection (Days Before Prediction Point of 3-months Post Diagnosis)	Second Infection (Days Before Prediction Point of 3-months Post Diagnosis)	First Infection (Days Before Prediction Point of 3-months Post Diagnosis)	Qualitative Description of Infection Distribution
**01**	25	36	50	61	Dense Recent Infections
**02**	200	240	250	300	Dense non-Recent Infections
**03**	10	100	300	500	Non-Dense Infections
**04**	15	150	177	900	Recent and non-recent Infections

Supplementary Figure 3. Infection date modelling features. Infection data was included from the microbiology data source and the drawing of a blood culture irrespective of the result was counted as an infection. No Infection data was used beyond the prediction point of 3-months post-diagnosis. **a,** List of infection date modelling features. The features detailed in the table were each calculated for the 3 look-back windows used in this work: 3 months, 1 year and 7 years. **b,** Example of linear and quadrative fitting features to quantify differences in infection distributions for patients with 4 past infections each. For example, infections that are 'dense and recent', 'dense but not recent', 'not-dense' and 'recent with non-recent infections' could be distinctly categorized using the coefficients of the fitted curves. In summary, the coefficients of the fitted slopes may therefore be used as features to describe different infection distributions and assess their effect if any on the risk level of a given patient. **c,** Example of how patients with 4 past infections each may have different time distributions. Linear and quadratic fitting in the form presented in (b) are able to quantitatively distinguish between the four distinct infection time distributions presented here.

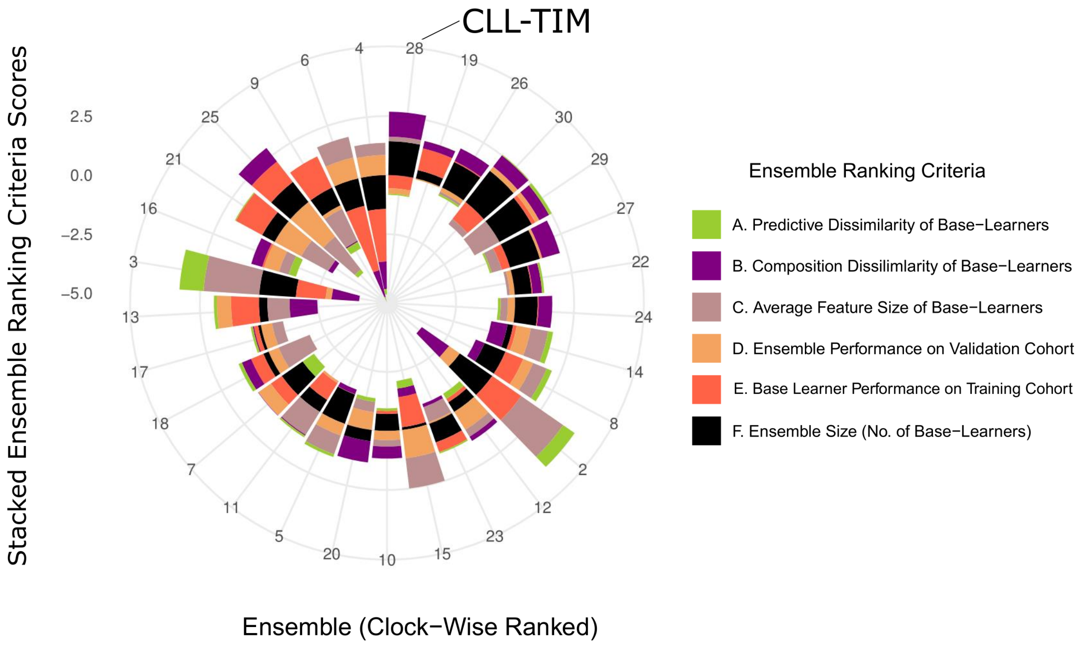

Supplementary Figure 4. Ensemble Scoring and Ranking. Ensemble ranking and selection of CLL-TIM. The 29 ensembles generated by our genetic algorithm were ranked according to an unweighted average of six generalization criteria (See Methods). CLL-TIM, an ensemble of 28 base-learners, is chosen as the highest ranked ensemble. Both training and validation cohorts were used for ranking.

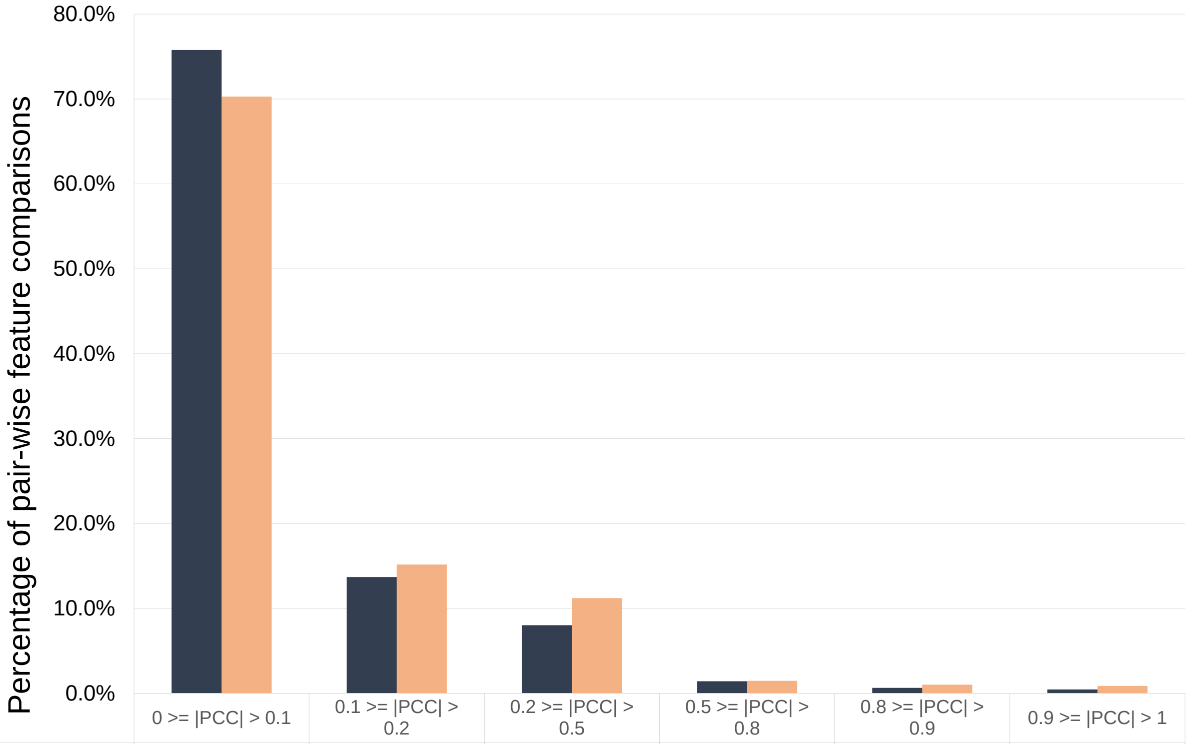

	0 >= |PCC| > 0.1	0.1 >= |PCC| > 0.2	0.2 >= |PCC| > 0.5	0.5 >= |PCC| > 0.8	0.8 >= |PCC| > 0.9	0.9 >= |PCC| > 1
Missing Data Imputation	75.8%	13.7%	8.0%	1.4%	0.6%	0.4%
On Non-Missing	70.3%	15.2%	11.2%	1.5%	1.0%	0.9%

Supplementary Figure 5. CLL-TIM feature redundancy analysis. For CLL-TIM's 228 features, we performed Pearson's Correlation Coefficient (PCC) on all possible pair-wise combinations. This analysis was performed on the 4149 patients in our internal Danish cohort and none of this information was used to build CLL-TIM. We used two methods for comparing the PCC of features with missing values: i) 'Missing Data Imputation': Missing data was imputed with median values and comparison made on all patients; ii) 'On Non-Missing': Correlation was calculated only on the subset of patients with non-missing values for the features compared. The highest correlations were observed between the date of tests for eosophilocytes, basophilocytes, lymphocytes, neutrophil count, leukocytes and hemoglobin; the dates of tests for blast cells, myelocytes and metamyelocytes; the values for leukocytes and lymphocytes; as expected due to these variables being included in the same medical order (differential count). Similarly, for missing value indicators of the different FISH status variables, a high correlation was seen. For these features, feature importance may be underestimated in our model. However, with 75% of all possible pair-wise feature correlations having |PCC|<0.2, we expect the underestimation of feature importance to be minimal. The low feature redundancy exhibited in CLL-TIM suggests that it uses complimentary information in features for its predictions.

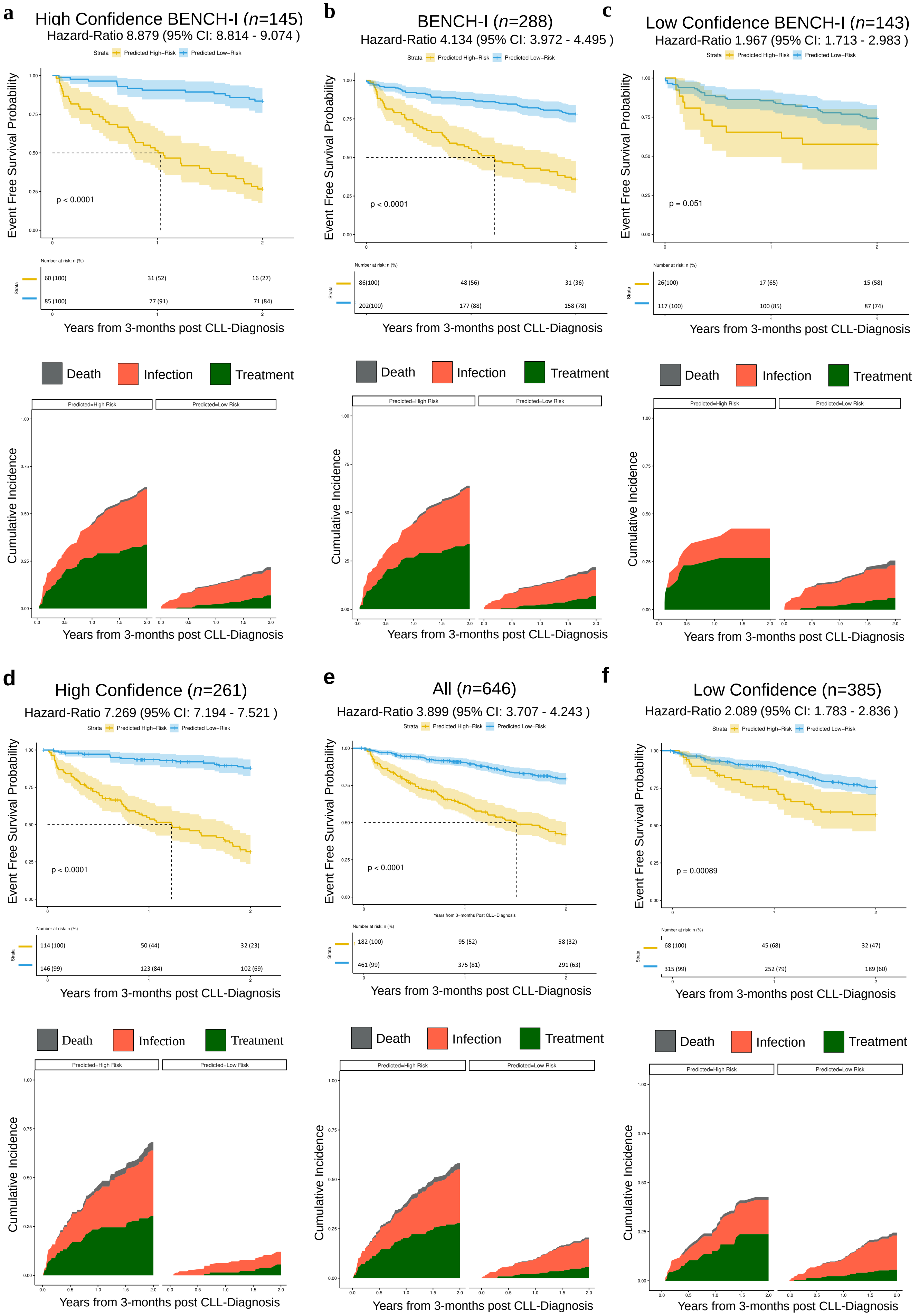

Supplementary Figure 6. Event free survival and cumulative incidence plots for CLL-TIM on internal test cohort. a, and **d,** CLL-TIM's predictions that were indicated as high-confidence (i.e. top 20% ranked as high-risk and top 30% ranked as low-risk) – referred to in main text as CLL-TIM (HC). **b,** and **e,** Predictions on entire cohort with no restriction of confidence – referred to in text as CLL-TIM. **c,** and **f,** Low confidence shows the remaining subset of CLL-TIM's predictions that were indicated as uncertain i.e. not high confidence. Confidence (value ranging from 0 to 1) was estimated using the average probabilistic output of all 28 machine learning algorithms used in CLL-TIM. For kaplan-meier curves, *p*-value is by log-rank test and infection, CLL treatment and death were all considered an event. Patients lost to follow-up were censored. BENCH-I – is the internal benchmark with patients having full CLL-IPI and a full 2-years follow-up post prediction point. Prediction point was at 3 months post CLL diagnosis. (a-c) show no censoring as for the BENCH-I cohort all patients had a full 2-year follow-up.

a

Protocol 29 ensembles per protocol	Description & Motivations	Training Target Outcome	Feature Modelling	Patient Data Look-back from Prediction Point
**ENS-COMP**	Ensembles generated using the data-driven protocol like CLL-TIM.	Treatment or Infection	Data-Driven	3 months / 1 year / 7 year
**ENS-COMP$_{DC}$ (Doctor's Choice)**	Ensembles using a set of features that have already been shown to be related to CLL prognosis. This enables us to compare the validity of a fully-data driven approach like CLL-TIM against that of hand-selected variables. The list of variables available for ENS-COMP$_{DC}$ were: lab test value and date modelling (Figure 1) of Lymphocytes , Leukocytes , Neutrophils, Platelets, Hemoglobin, Reticulocytes, Lactate dehydrogenase, C-reactive protein, Albumin, Creatinine, Immunoglobulin A, Immunoglobulin G, Immunoglobulin M, Haptoglobin, BOW Modelling (Figure 1) of Microbiology findings (including blood culture findings) , infection data modelling , and Baseline variables: Age, Gender, Binet Stage, beta-2-microglobulin, CD38, IGHV unmutated, del(13q), tri(12), del(11q), del(17p), Familiar CLL, and ECOG/WHO Performance Status. Therefore, the set of hand-selected variables underwent the same feature encoding and feature selection processes as those for CLL-TIM .	Treatment or Infection	Pre-selected variables only	3 months / 1 year / 7 year
**ENS-COMP$_{3m}$ (3-months Look-back)**	Ensembles using only data available within the first 3-months of CLL diagnosis. Given that CLL-TIM uses data pre-CLL diagnosis, this enables us to quantify the effect of not having such patient data available	Treatment or Infection	Data-Driven	3 months
**ENS-COMP$_{CLL-IPI}$ (CLL-IPI Variables)**	Ensembles using only CLL-IPI variables and thus enabling us to assess the discriminatory power of CLL-IPI variables in the context of an ensemble model.	Treatment or Infection	CLL-IPI variables only	3 months
**CLL-IPI 4+**	CLL-IPI Score 4-10 as high risk vs CLL-IPI Score 0-3 as low risk.	Developed for prediction of Overall Survival (OS) and validated for time-to-first-treatment (TTFT)	-	3 months
**CLL-IPI 2+**	CLL-IPI Score 2-10 as high risk vs CLL-IPI Score 0-1 as low risk.	OS/TTFT	-	3 months

b **c**

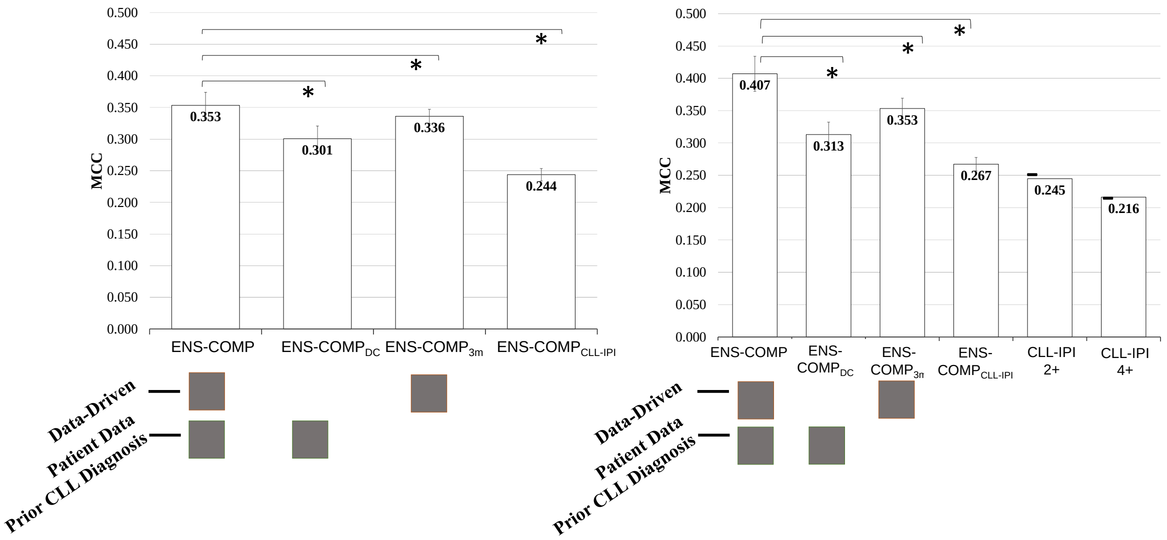

Supplementary Figure 7. Ensemble benchmark models. a, Summary of ensemble benchmark models. The protocol for generation of CLL-TIM, is both data-driven and uses data several years prior to CLL diagnosis (ENS-COMP). For ENS-COMP, 29 ensembles were generated from which CLL-TIM was selected. ENS-COMP$_{DC}$ (Doctor's Choice) is a protocol identical to ENS-COMP except that the initial set of variables were pre-selected by two of our experienced physicians. ENS-COMP$_{3m}$ (3-months look-back) is a protocol identical to ENS-COMP except that look-back was restricted to 3-months from prediction point – and hence no patient data prior CLL diagnosis was used for modeling. ENS-COMP$_{CLL-IPI}$ is a protocol identical to ENS-COMP except that the initial set of variables were limited to CLL-IPI variables only. CLL-IPI 4+ and CLL-IPI 2+ use scores directly from the CLL-IPI score. With the exception of their unique specifications, all ensemble models were generated using the same protocol as CLL-TIM. Therefore, the process of base-learner generation, ensemble generation, ensemble scoring and selection of the final ensemble, was run independently for each protocol. Training target outcome refers to the outcome the ensembles were originally trained to predict. All ensembles were then analyzed on their ability to predict the composite outcome i.e. outcome of infection or treatment within the next 2-years from prediction point. The prediction point was defined as 3-months post diagnosis. **b,** Results on subset of patients in internal test cohort with a full 2-year follow-up (*n*=530). **c,** Results on subset of patients in internal test cohort with a full 2-year follow-up and full CLL-IPI variables (BENCH-I, *n*=288). Results shown in b-c are for the average matthew's correlation coefficient (MCC) of the 29 ensembles (base-learner sizes 2-30) generated from each protocol. *P*-values were calculated using one-tailed wilcoxon signed rank test on the difference in 29 mean MCCs between ENS-COMP and ENS-COMP$_{DC}$, ENS-COMP$_{3m}$ and ENS-COMP$_{CLL-IPI}$. Pairing was performed with ensembles size. * is for $p<0.05$, - is for uncalculatable p-values since CLL-IPI are not ensembles.

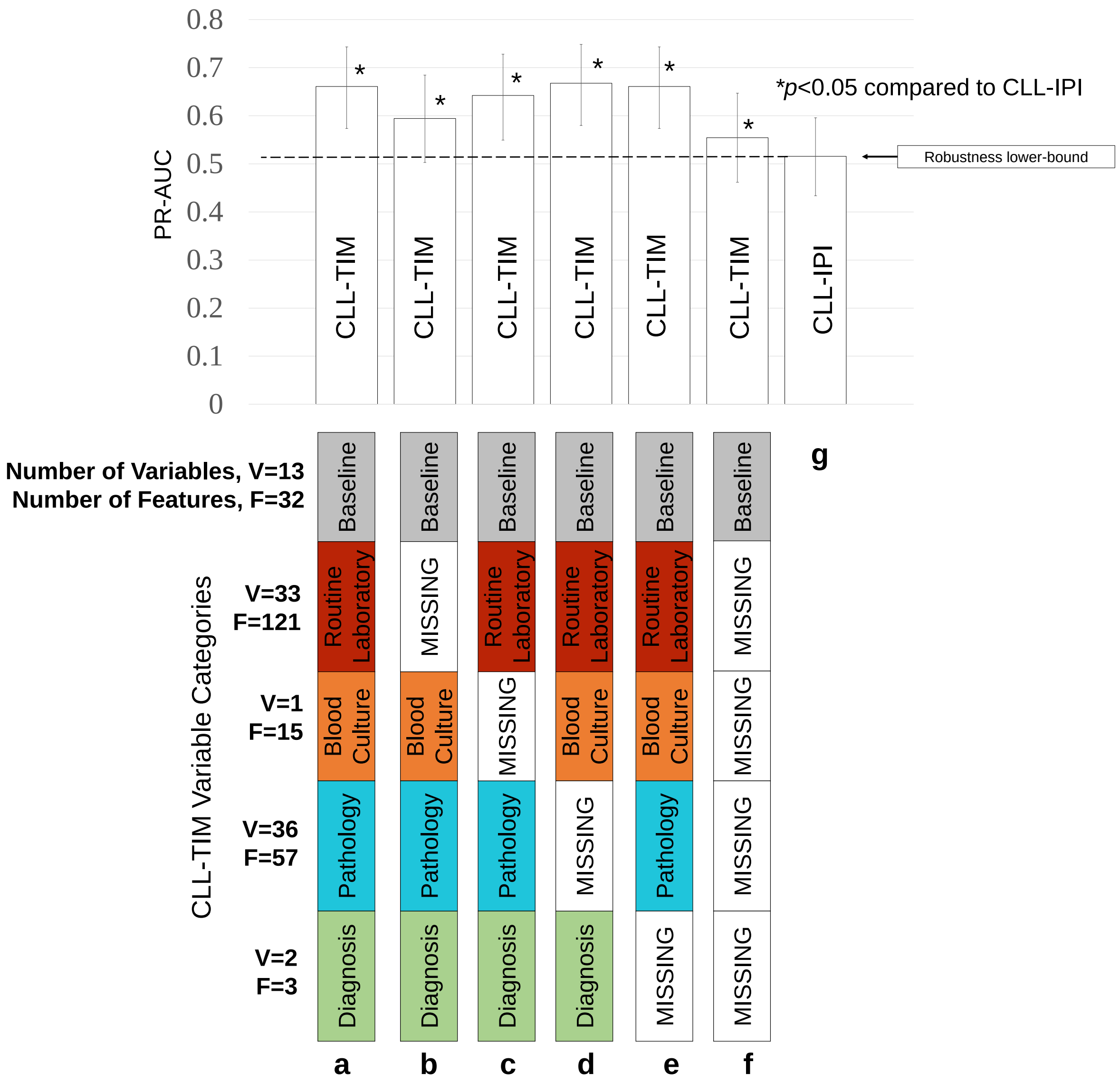

Supplementary Figure 8. Robustness Simulations on internal test cohort. CLL-TIM uses:13 Baseline variables (encoded to 32 features); 33 Routine laboratory test variables (121 features); 1 Blood culture variable (15 features); 36 Pathology variables (57 features) and 2 diagnosis variables (3 features) - full list provided in Supplementary Table 1. **a,** As our reference value we use CLL-TIM's PR-AUC on BENCH-I (*n*=288). Performance of CLL-TIM on BENCH-I with **b,** all routine laboratory variables set to missing **c,** no infection data **d,** all pathology data set to missing **e,** all diagnosis data set to missing **f,** no data except for baseline variables. **g,** Our lower-bound PR-AUC value for claiming robustness of CLL-TIM under missing data conditions is the performance of CLL-IPI on BENCH-I. Patients in in the internal test cohort had an average missing feature rate per base-learner of 3%-48%, and hence the aforementioned missing data simulations were done in addition to the inherent missing data already present. Confidence intervals were generated using predictions on 5000 bootstrapped datasets sampled with replacement from the internal test cohort. Comparison of **b-f** to **g** were performed using a one-tailed Wilcoxon (* is $p<0.05$) signed-rank test on the difference in means of PR-AUC over the 5000 bootstrapped datasets. PR-AUC: is the precision-recall area-under-curve. BENCH-I (*n*=288) is a subset of the internal test cohort (*n*=646) were patients have full CLL-IPI and full 2-year follow up post the prediction point of 3-months post-diagnosis. *$p<0.05$ compared to CLL-IPI

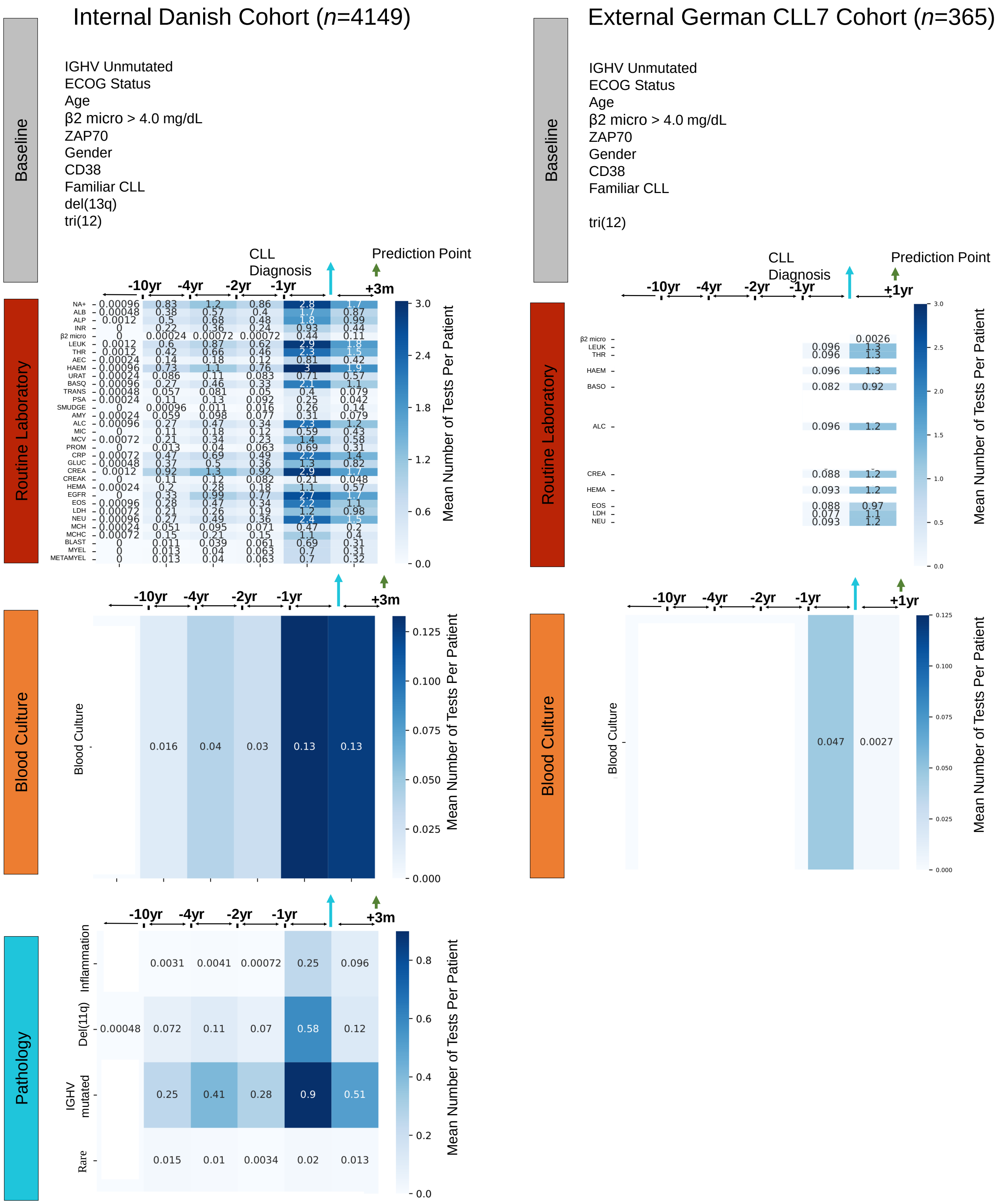

Supplementary Figure 9. Missing data conditions in external cohort compared to internal cohort. Baseline characteristics for both cohorts are found in Table 1. CLL-TIM uses:13 Baseline variables; 33 Routine laboratory test variables; 2 Diagnosis variables; 36 Pathology variables and blood cultures (Figure 2 and Supplementary Data 1). Shown here are all 33 routine laboratory variables used by CLL-TIM and for simplicity, for all other variables, only those that were part in more than 10% of CLL-TIM's base-learners are shown; 11 Baseline variables; 4 Pathology variables and blood cultures. For each variable, we calculated the mean number of times they show up in a CLL patient's history. This, in several time windows preceding our prediction point of 3 months post-diagnosis, and those preceding CLL diagnosis: 1 year prior CLL Diagnosis; 1 to 2 years prior CLL Diagnosis, 2 to 4 years prior CLL diagnosis; 4 to 10 years prior CLL-Diagnosis, and more than 10 years prior CLL-Diagnosis. The external German CLL7 cohort had no data from pathology and diagnosis reports, and only a few variables prior to CLL diagnosis. In addition, patients had data for only 11 of CLL-TIM's 33 routine laboratory tests. Apart from del(13q) all baseline data was available. The external CLL7 cohort thus enabled us to assess the effect of having few laboratory tests without data points prior CLL diagnosis and no sufficient data for pathology and infection history.

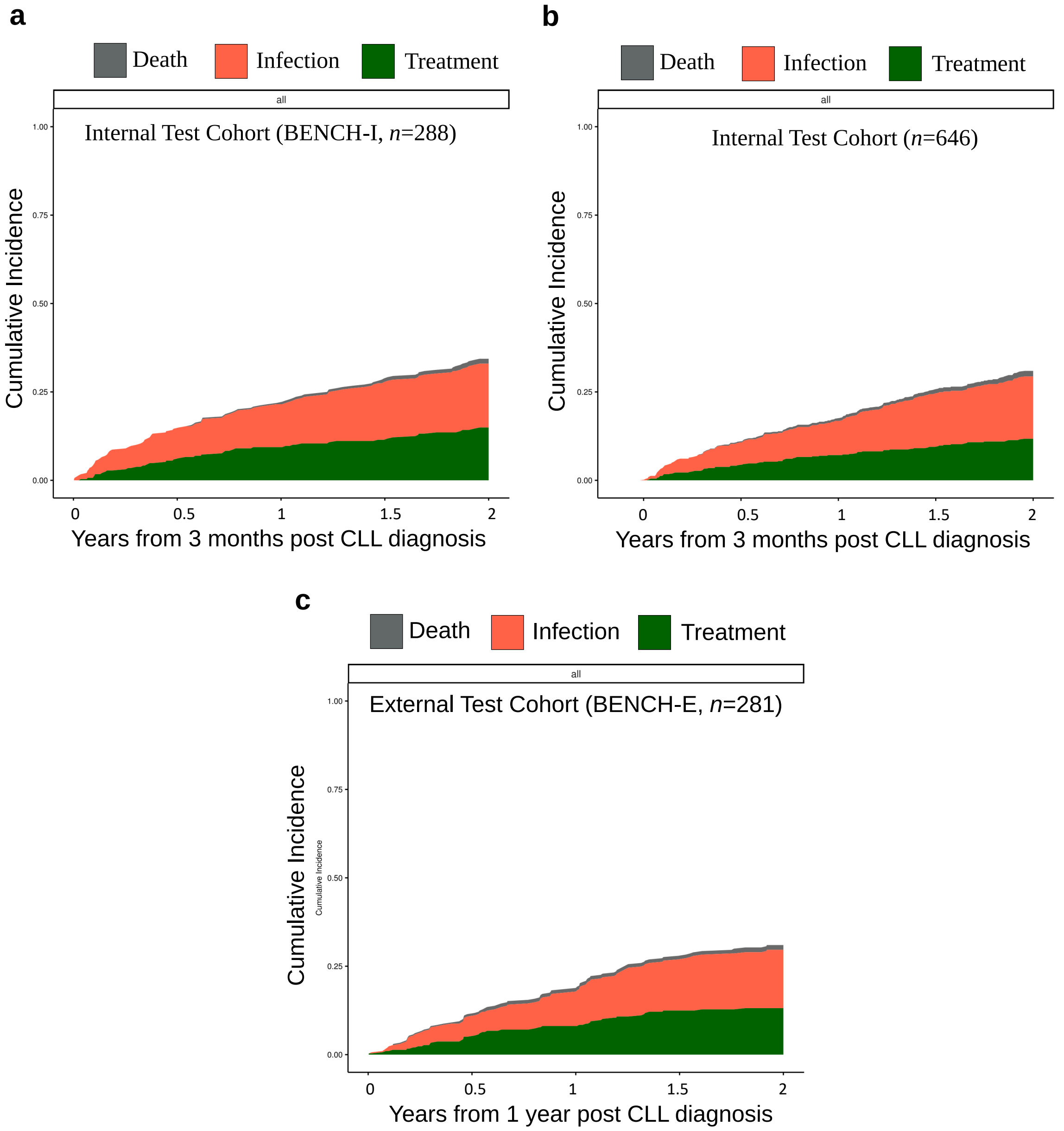

Supplementary Figure 10. Cumulative incidence for the Danish internal test cohort and the German CLL7 external cohort. **a,** Internal benchmark (BENCH-I) refers to subset of cohort for which patients have full CLL-IPI and full 2-year follow up in our Danish internal test cohort. **b,** Entire Danish internal test cohort. **c,** External benchmark (BENCH-E) refers to subset of cohort for which patients have full CLL-IPI and full 2-year follow up in the German external CLL7 study. Prediction point for internal cohorts was at 3 months post CLL diagnosis and that for external cohort was at 1 year post CLL diagnosis. Minimal data prior to CLL diagnosis was available in the external cohort, and hence to allow for sufficient laboratory tests to be available for patient predictions, the prediction point for the external test cohort was thus extended to 1 year.

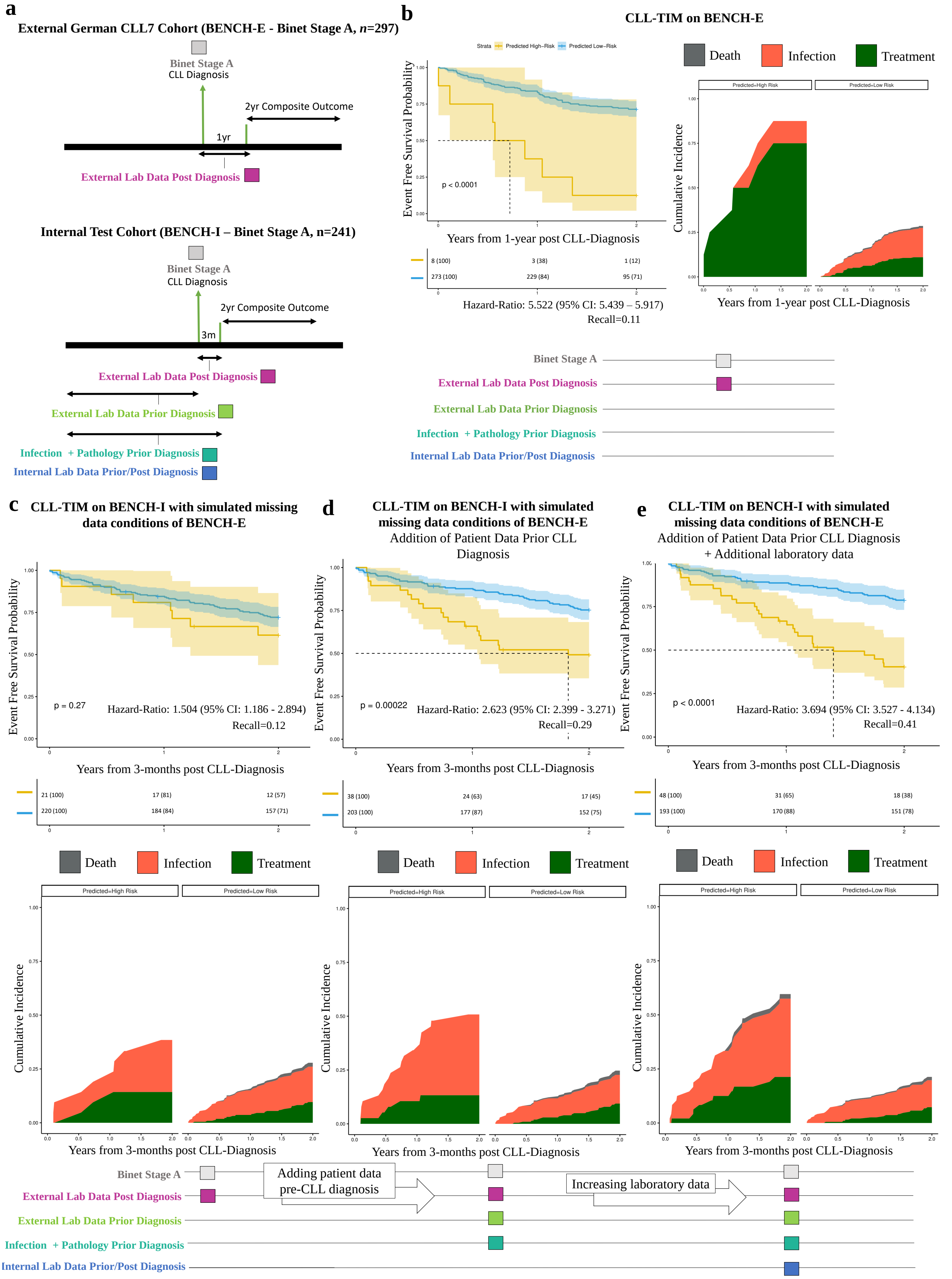

Supplementary Figure 11. Internal test cohort simulations of missing data conditions of CLL7 external cohort. a, Summary of data and prediction time-lines on external (BENCH-E, *n*=281) and internal benchmark test cohorts (BENCH-I, *n*=288). BENCH-I and BENCH-E refer to subsets of the internal and external cohorts respectively were patients had full CLL-IPI and a full 2-year follow-up. All patients in CLL7 cohort were Binet stage A, no patient data prior to CLL diagnosis was available and laboratory data was only available for hemoglobin, erythrocyte count, leukocytes, neutrophilocytes, basophilocytes, eosinophilocytes, lactate dehydrogenase, platelets, absolute lymphocyte count and creatinine (i.e 11 of the 33 laboratory tests used by CLL-TIM). Reticulocytes, immunoglobulin A, immunoglobulin G, immunoglobulin M were available but not part of CLL-TIM and hence not part of this analysis. Baseline characteristics for internal and external cohorts are found in Table 1. **b,** CLL-TIM predictions on BENCH-E. **c,** Given the reduction in Recall and prediction of infection events prior to treatment on the BENCH-E, we simulated missing data conditions of BENCH-E on BENCH-I and re-ran CLL-TIM. For this, conditions necessitated all patients to be Binet Stage A, and to restrict laboratory data of the 11 laboratory tests available in BENCH-E to data post CLL diagnosis. **d,** Using the same conditions in (c) we also added data prior CLL diagnosis for the 11 laboratory tests, and also pathology and infection data. CLL-TIM was re-run under these new conditions. **e,** Using the same conditions in (d) data for all 33 laboratory tests used by CLL-TIM were added and CLL-TIM re-run. All predictions shown here put no restriction to high-confidence predictions. Even though CLL-TIM's high-confidence predictions show better discrimination ability, the motivation of this analysis was to compare relative performance with various missing data conditions on the benchmark cohorts.

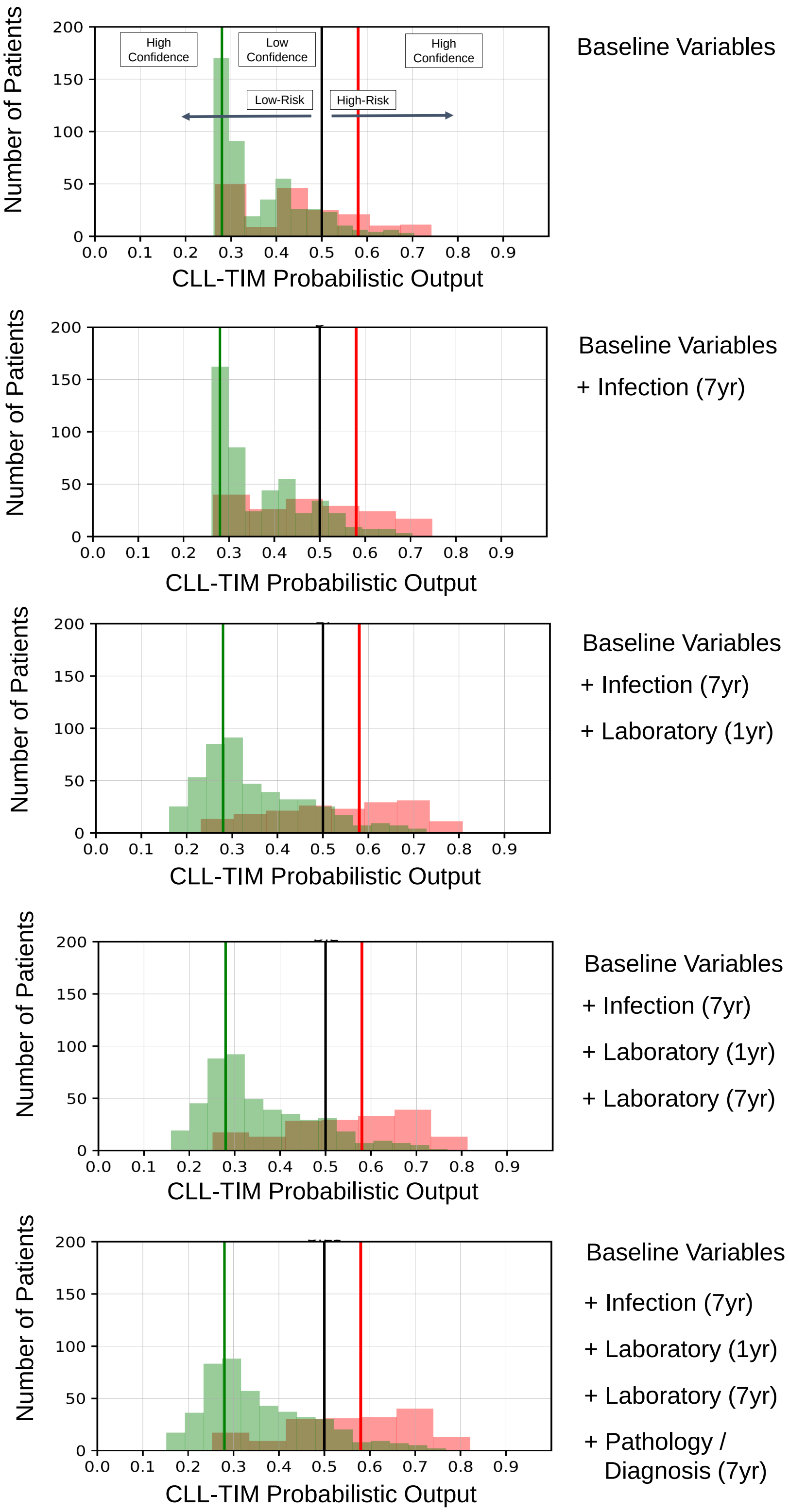

Supplementary Figure 12. Distribution of CLL-TIM's probabilistic output on addition of patient data. Data is shown for patients in the internal Danish test cohort (*n*=646) and therefore they were not seen during CLL-TIM's training. Red distribution - truly high-risk patients, green distribution – truly low-risk patients. Overlaps of the two distributions represent misclassifications.We performed five rounds of CLL-TIM predictions on each patient, where in each round we added more data starting from baseline, adding infection history, laboratory variables up to a one year prior to CLL diagnosis, laboratory variables up to a seven years prior to CLL diagnosis and finally the addition of Pathology and Diagnosis variables. Probabilistic output thresholds for high-confidence (0.28>*P*>0.58) predictions were derived from the validation cohort.

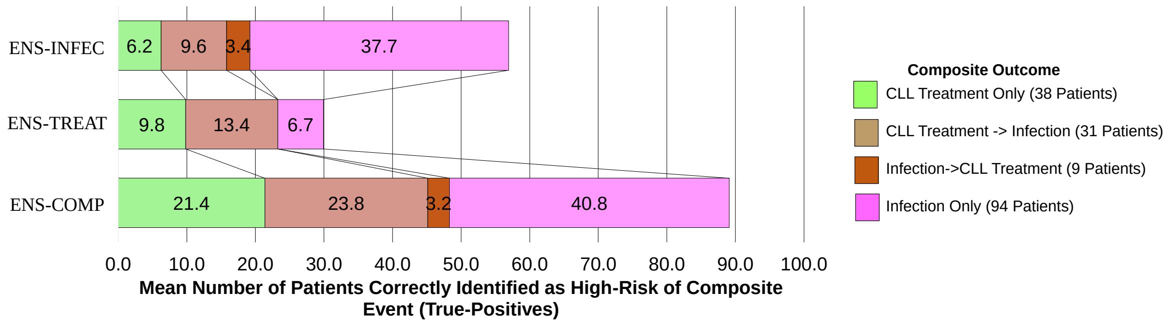

Infection Only[a]		ENS-COMP (TP=40.8)	ENS-TREAT (TP=6.7)	ENS-INFEC (TP=37.7)
	ENS-COMP	x	2.24E-06	**0.07**
	ENS-TREAT		x	1.16E-06
	ENS-INFEC			x

Infection->CLL Treatment[b]		ENS-COMP (TP=3.2)	ENS-TREAT (TP=0)	ENS-INFEC (TP=3.4)
	ENS-COMP	x	2.24E-06	**0.1**
	ENS-TREAT		x	2.51E-06
	ENS-INFEC			x

CLL Treatment Only[c]		ENS-COMP (TP=21.4)	ENS-TREAT (TP=9.8)	ENS-INFEC (TP=6.2)
	ENS-COMP	x	2.38E-06	2.47E-06
	ENS-TREAT		x	2.10E-04
	ENS-INFEC			x

CLL Treatment->Infection[d]		ENS-COMP (TP=23.8)	ENS-TREAT (TP=13.4)	ENS-INFEC (TP=9.6)
	ENS-COMP	x	2.23E-06	2.41E-06
	ENS-TREAT		x	9.56E-05
	ENS-INFEC			x

Infection Prior to CLL Treatment[a+b]	ENS-COMP (TP=44.0)	ENS-TREAT (TP=6.7)	ENS-INFEC (TP=41.1)
ENS-COMP	x	2.09E-06	**0.08**
ENS-TREAT		x	2.52E-06
ENS-INFEC			x

CLL Treatment Prior to Infection[c+d]	ENS-COMP (TP=45.1)	ENS-TREAT (TP=23.3)	ENS-INFEC (TP=15.8)
ENS-COMP	x	2.41E-06	2.50E-06
ENS-TREAT		x	7.33E-05
ENS-INFEC			x

Supplementary Figure 13. Statistical testing for modelling infection and CLL treatment separately and jointly. Results shown for patients with full 2-year follow-up on internal test cohort (*n*=530). P-values are for the two-tailed Wilcoxon signed-rank test of the number of true-positive (TP) predictions of the 29 ensembles (2-30 base-learners) generated in each of the 3 protocols (See Supplementary Table 5). Pairing was performed using ensemble size. The protocols differ in the target outcome they were trained to predict: ENS-INFEC predict infection as a first event; ENS-TREAT predict treatment and ENS-COMP predict the composite outcome. All protocols were tested for their ability to predict the four mutually exclusive possibilities of the composite outcome: ‘Infection Only’ for patients who only had an infection in the 2-year follow-up; ‘Infection->CLL Treatment’ for patients who had an infection followed by initiation of CLL treatment in the 2-year follow-up; ‘CLL Treatment Only’ for patients who only had CLL treatment in the 2-year follow-up; ‘CLL Treatment>Infection’ for patients who had CLL treatment followed by an infection in the 2-year follow-up. Also compared is: ‘Infection Prior to Treatment’, which includes ‘Infection’ and ‘Infection>CLL treatment’ and; ‘Treatment Prior to Infection’ which includes ‘CLL Treatment’ and ‘CLL Treatment->Infection’. In blue are highlighted instances where no significant difference (p>0.05) was found in the number of true-positives predicted by each set of ensembles within the compared protocols.

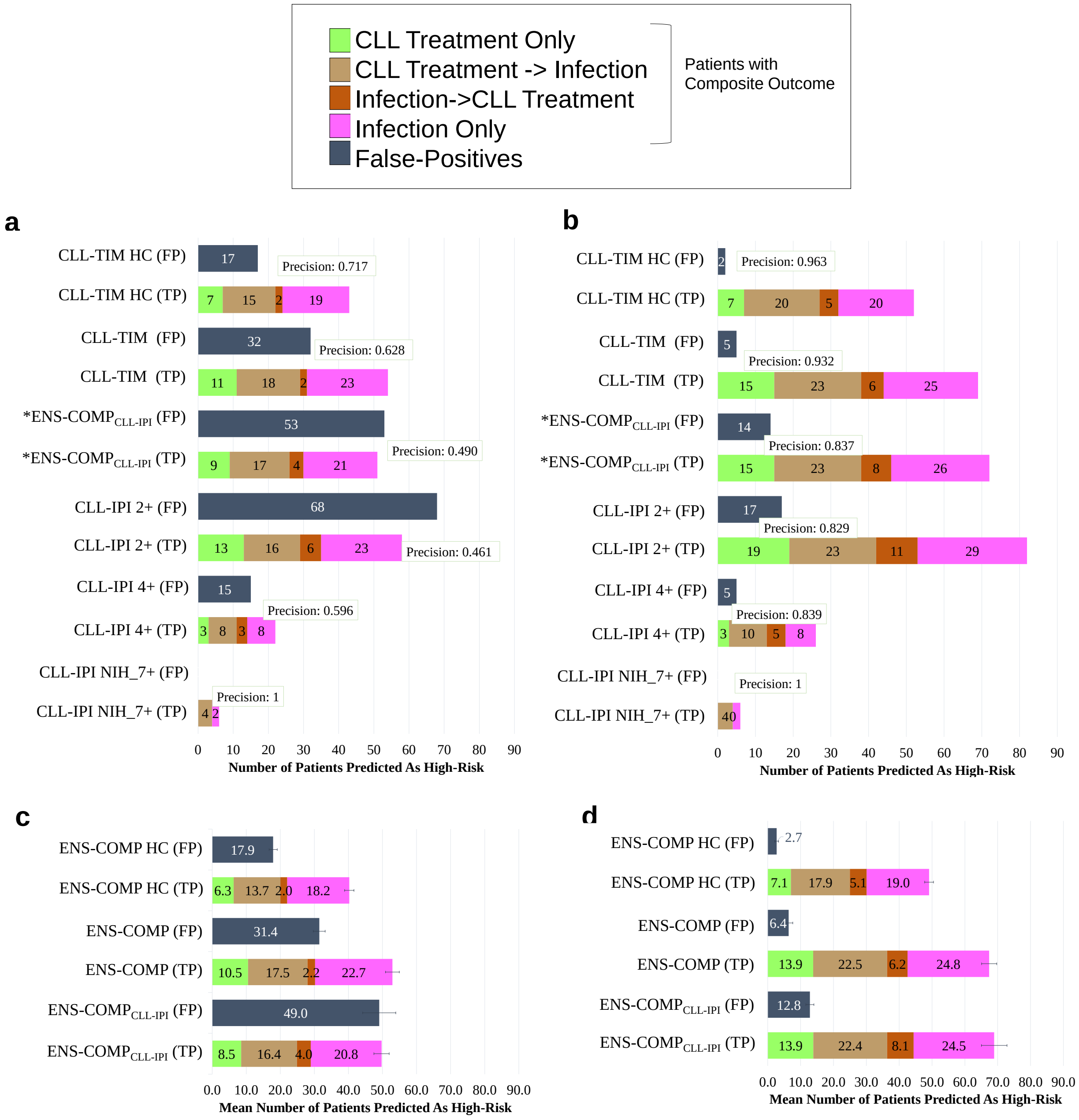

Supplementary Figure 14. Detection of Infection prior to Treatment with CLL-TIM and CLL-IPI. True-positive predictions are separated into the four mutually exclusive possibilities of the composite outcome: Treatment only – patient initiated CLL treatment without prior infections; Treatment->Infection – patients initiated CLL treatment and developed an infection post-treatment; Infection – patients had an infection but did not start CLL treatment; Infection->Treatment patients had an infection followed by initiation of CLL treatment. All events considered were restricted to the 2-year or 5-year predictive window. Predictions were assessed on BENCH-I Cohort – Subset of patients in test cohort with full CLL-IPI variables available and a full 2-years / 5-years follow-up. **a,** CLL-TIM vs CLL-IPI on $\text{BENCH-I}_{\text{2-year}}$ *n*=288 and **b,** CLL-TIM vs CLL-IPI on $\text{BENCH-I}_{\text{5-year}}$ *n*=203. CLL-TIM's High-Confidence (HC) predictions are on a subset of the cohort i.e. on those patients for which CLL-TIM was more confident in (without knowledge of the true outcome). $\text{*ENS-COMP}_{\text{CLL-IPI}}$ is the top ensemble model trained to predict the composite outcome using CLL-IPI variables. The protocol used for developing $\text{*ENS-COMP}_{\text{CLL-IPI}}$ was identical to CLL-TIM's protocol except for the former being restricted to CLL-IPI variables only. CLL-IPI 2+ and 4+: patients with CLL-IPI score of 4 or above are considered as high-risk. CLL-IPI NI_4+: Same as CLL-IPI 4+ but with patients in the CLL-IPI intermediate-risk category (2-3) removed from analysis. CLL-IPI NIH_7+: patients with CLL-IPI score of 7 or above are considered as high-risk, and patients with CLL-IPI intermediate and high-risk groups (2-6) removed from analysis. These removals had the effect of considering CLL-IPI predictions at the extremes and thus the highest and lowest risk patients according to the CLL-IPI score. **c,** Ensemble models trained on all features vs CLL-IPI features on 2-year outcome. **d,** Ensemble models trained on all features vs CLL-IPI features on 5-year outcome. ENS-COMP refers to 29 ensemble models (2-30 base-learners) trained to predict the composite outcome generated using the same protocol as CLL-TIM. CLL-TIM is one of the ensembles in this set. $\text{ENS-COMP}_{\text{CLL-IPI}}$ refers to 29 ensemble models (2-30 base-learners) trained to predict the composite outcome generated using the same protocol as CLL-TIM but with restriction to using only CLL-IPI variables. $\text{*ENS-COMP}_{\text{CLL-IPI}}$ is one of the ensembles in this set (See Supplementary Fig. 7 for summary). Error-bars indicate standard deviation across the predictions of 29 ensemble models. TP – True-Positive Predictions. FP- False Positive Predictions.

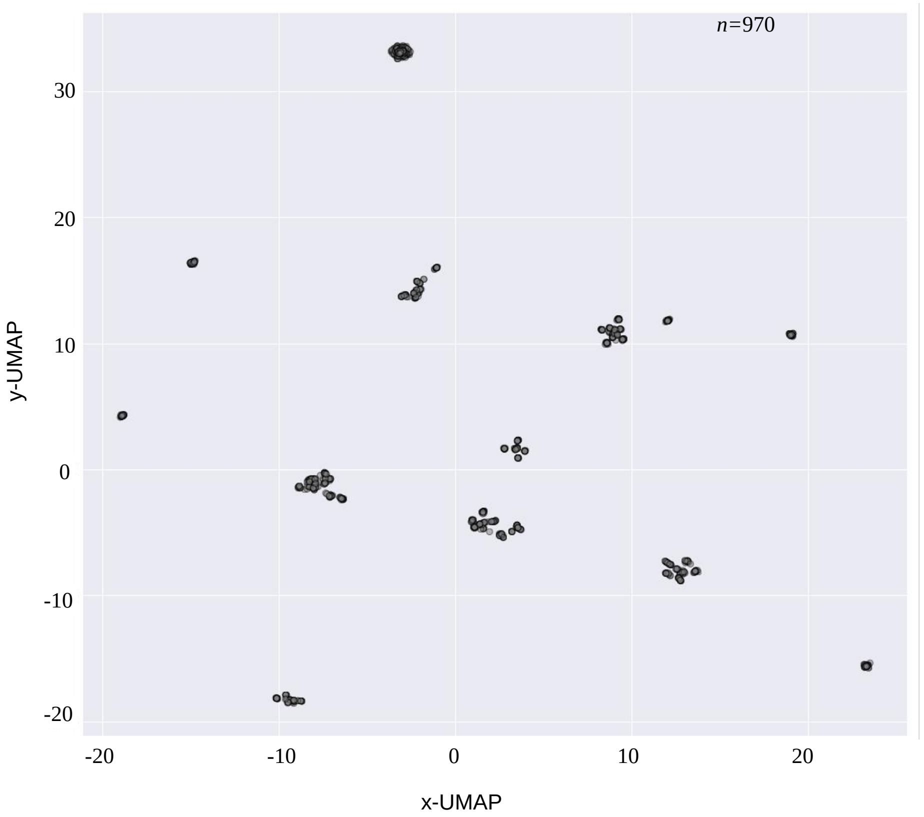

Supplementary Figure 15. UMAP clustering for personalized high-risk factors in CLL-TIM. First we generated the top 3 personalized risk-factors for each of the 970 patients with a composite outcome in the Danish cohort. For this we used Shapley Additive explanations (SHAP) over the 28 base-learners of CLL-TIM. On these, we performed Uniform Manifold Approximation and Projection (UMAP) clustering of the top 3 personalized high-risk factors. UMAP clustering was performed using umap python library. We set the number of neighbors to 30 as this parameter was also 30 for clusters generated using t-Distributed Stochastic Neighbor Embedding (t-SNE). Other parameters were left as their default.

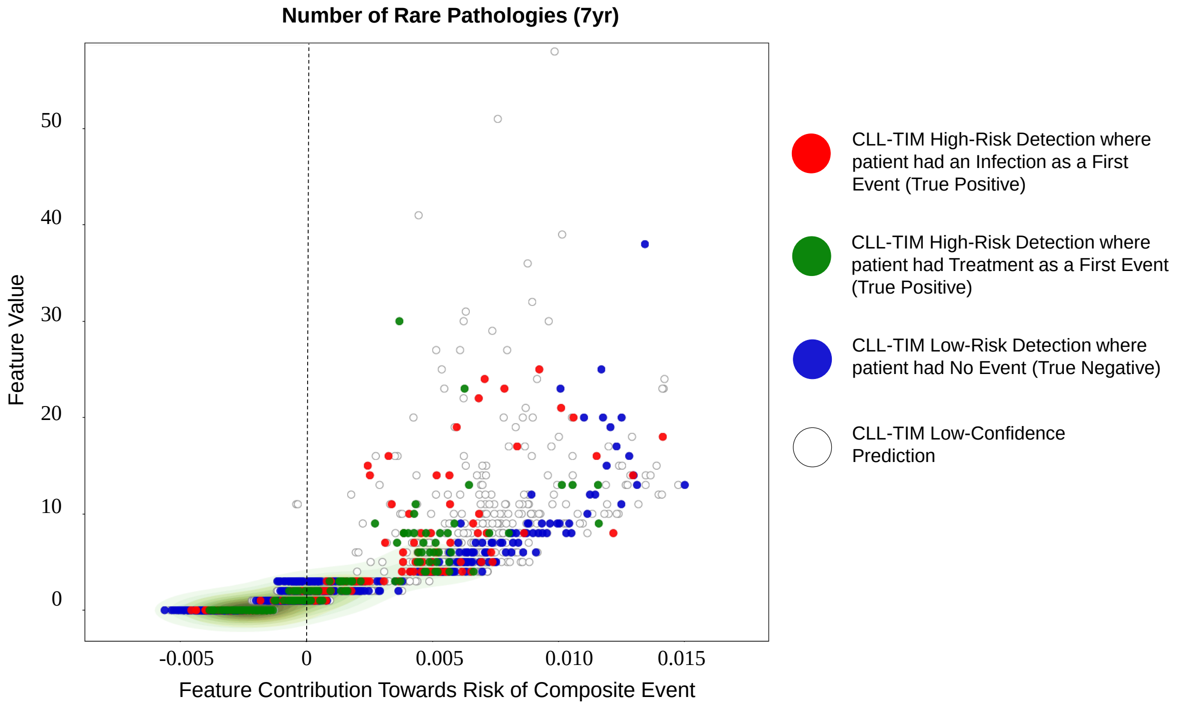

Supplementary Figure 16. **Variable-to-risk mapping learnt by CLL-TIM for Rare Pathology.** Rare pathologies were defined as those pathologies that were found in less than 1% of the CLL population. There were 1419 rare pathologies in total and our physicians went through a random selection from the list and confirmed the presence of several comorbidity related events. These however also included non-comorbidity related events and several words that point to similar findings for the same patient. For the high number of rare pathologies, we hypothesized that comorbidity is likely to be represented, at least in part, by these. This may or may not have an effect on risk of treatment and infection. To assess this, we generated a variable-to-risk mapping for the 'Rare Pathology'. Data-points are for true-positive (red and green) and true-negative (blue) patient predictions of CLL-TIM, with high-confidence. Feature contributions were extracted using SHAP over CLL-TIM's 28 base-learners. CLL-TIM models an increase in the number of rare pathologies with a higher risk of infection or treatment. Specifically, above *circa.* four rare pathologies, as the number of rare pathologies increases, there is a continuous increase in risk of infection or treatment. CLL-TIM limits the contribution of 'Rare Pathology' when the number of words is higher than *circa.* 20, thereby not overestimating the risk contribution in these cases. It should also be noted that the range of contribution of 'Rare Pathology' to CLL-TIM's probabilistic output (x-axis: -0.005 to 0.015), is minor compared to the risk factors presented in Fig 8.

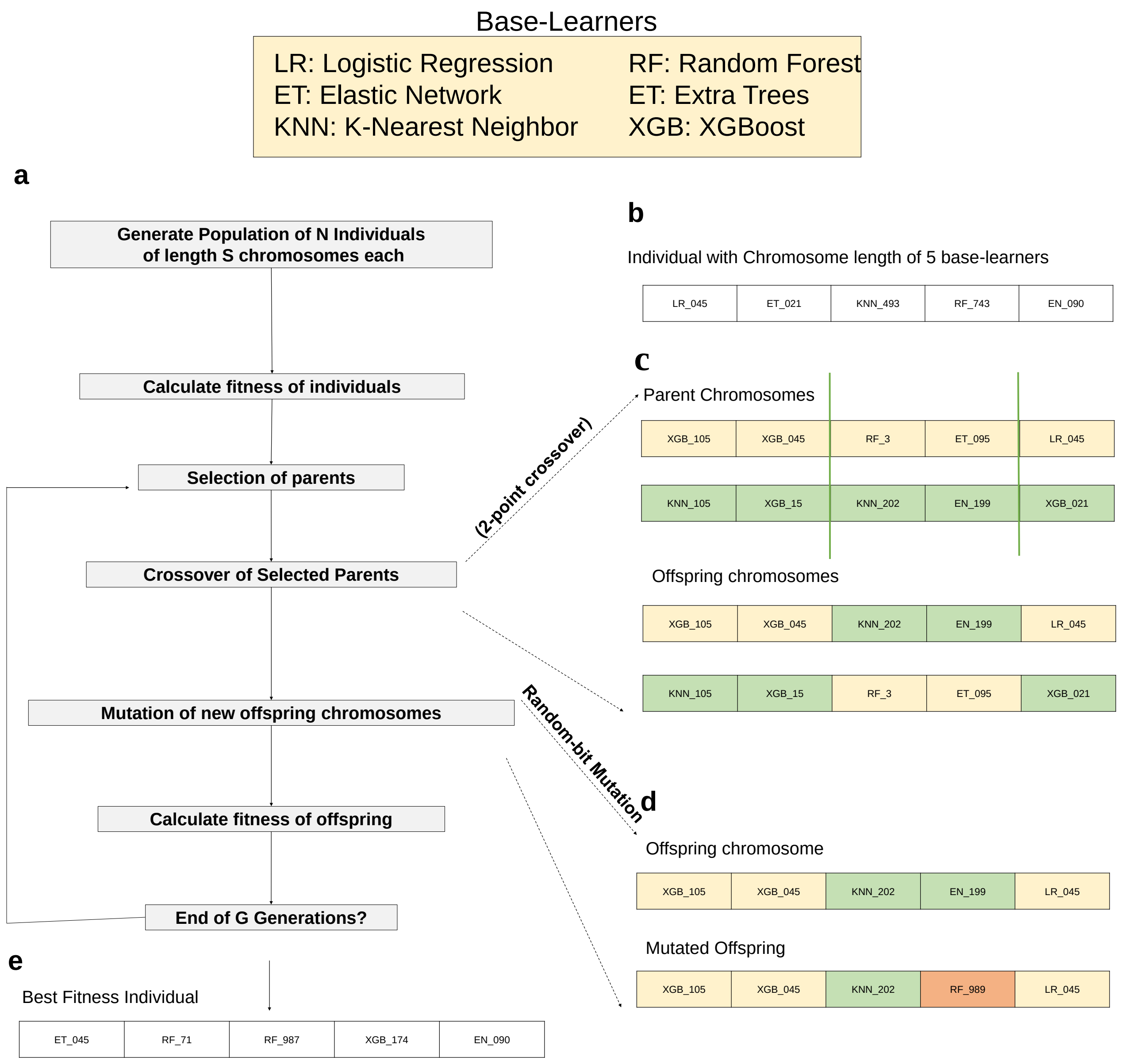

Supplementary Figure 17. Genetic algorithm ensemble generation. Genetic Algorithms (GA) are a class of meta-heuristic optimization algorithms inspired by natural evolution. In this work a GA was designed to optimize the selection of a subset of *S* base-learners from a set of 2000**. a,** An initial population of *N*=10,000 individuals were randomly generated with a chromosome length *S*, where *S* is the size of the ensemble. Fitness was calculated as the Matthew's Correlation Coefficient (MCC) of the ensemble on the validation cohort. Tournament selection of size 3 was performed for the selection of parents. Namely, 3 individuals were randomly selected, and the individual with the highest fitness is chosen as a parent. After all parents were selected, cross-over and mutation was performed to generate a set of 10,000 new offspring. The process was repeated for *G*=50 generations, by the end of which, the highest fitness individual is the final ensemble of size *S*. GA was run 29 times to generate ensembles for size 2-30 base-learners. **b,** Each individual is a set of base-learners forming through the average of their predictions, an ensemble prediction. The base-learners were pre-generated using training cohort data. **c,** 2-point cross-over was applied for offspring generation. Two cross-over points are randomly generated, and the new-offspring share alternating parts from the chromosomes of their parents. The probability that an offspring is produced by crossover was set to 0.5. **d,** With a probability of 0.05 an offspring is produced by a mutation where one base-learner is changed at random. **e,** The optimal ensembles of size *S*, is the best fitness individual by the end of 50 generations. The DEAP (Distributed Evolutionary Algorithms in Python) toolbox was used to develop the GA. Parameters, which are not mentioned, are assumed to be the default value for the given functions in DEAP.

Supplementary Table 1. Modeling of Lab Test Values and Dates. Seventeen feature encodings were designed to encode information in the 183 routine laboratory tests. No lab test data was used beyond the prediction point of 3-months post-diagnosis. The features described in the table were each calculated for the 3 look-back windows used in this work of 3 months, 1 year and 7 years. Features calculated on 'test values' summarize lab test values for tests that take place within each of the three look-back windows, irrespective of the point in time within the window they occurred. Features calculated on 'test dates and values' take into account both values and dates of lab test within each of the three look-back windows. For this group, least-squares fitting of linear and quadrative curves on the lab test values is performed. This enabled us to calculate rates of change and use estimations of future lab test values based on the respective fit as features. Features calculated on 'test dates' use only the date of a test, irrespective of the test outcome. This enabled us to model the clinicians' decision to test and the information withheld within the timing of such events.

Features Calculated on	Routine Laboratory Test Features	Aim & Description
**Test Values**	Value Mean	Mean Value
**Test Values**	Value SD	Standard Deviation of Values
**Test Values**	Value Max	Maximum Value
**Test Values**	Value Min	Minimum Value
**Test Values**	Value Median	Median Value
**Test Values**	Value Skewness	Captures whether values are more numerous in the high range vs the lower range, or symmetrical
**Test Values**	Value Kurtosis	Captures whether values built up to a maximum progressively or sharply
**Test Dates and Values**	Lab Test Value LinearFit_coefa (y=ax+b)	Rate of Change and Direction of Values
**Test Dates and Values**	Lab Test Value LinearFit_coefb (y=ax+b)	Estimated Value at Prediction Point (x=0)
**Test Dates and Values**	Lab Test Value QuadraticFit_coefa ($y=ax^2+bx+c$)	Steepness and Direction of Test Values
**Test Dates and Values**	Lab Test Value QuadraticFit_coefb ($y=ax^2+bx+c$)	Estimated Rate of Change at Prediction Point (x=0)
**Test Dates and Values**	Lab Test Value QuadraticFit_coefc ($y=ax^2+bx+c$)	Estimated Value at Prediction Point
**Test Dates**	No of Instances	Number of Test Taken
**Test Dates**	Date Mean	Recentness of Test Dates
**Test Dates**	Date SD	Spread of Testing Dates
**Test Dates**	Date Earliest	Days Since Earliest Test
**Test Dates**	Date Last	Days Since Last Test

Supplementary Table 2. Variables and Feature Encodings Summary. Baseline variables (Except for age) were one-hot-encoded to account for missing values (see Supplementary Table 25 for list of Baseline variables). Bag-Of-Words representation was used for pathology, diagnosis, microbiology and blood culture findings. Twelve features capture changes of routine lab values over time and 5 features capture changes in time irrespective of result (see Supplementary Table 1 for designed features). For blood cultures 16 time-distribution features are designed. For this, any blood culture irrespective of the finding was used (see Supplementary Fig. 2 for designed features). Three look-back windows of 3 months, 1 year and 7 years were used. Data cleaning involved the removal of single-valued encodings variables with 98% correlation.

Data Source	Variables	Encodings					
		One-Hot	Bag-Of-Words	Changes in Values over Time	Time of Test	Look-backs	Total (Derivation)
Baseline	13	38	-	-	-	-	38
Pathology	153	-	1	-	-	3	459 (153*3)
Diagnosis	216	-	1	-	-	3	648 (216*3)
Routine Lab	183	-	-	12	5	3	9333 (183*12*3+183*5*3)
Microbiology	46	-	1	-	-	3	138 (46*3)
Microbiology (Blood Culture)	9	-	1	-	16	3	75 (9*3+1*16*3)
	620 (Total)					Total	10691
						Total (after data cleaning)	7288

Supplementary Table 3. Base-Learner hyper parameters and feature selection methods. K-Nearest Neighbors (KNN), Logistic Regression (LR), Elastic Network (EN), Perceptron, Random Forest (RF), Extremely Randomized Trees - Extra Trees (ET), and Extreme Gradient Boosting – XGBoost (XGB). Parameter explanations may be found in Scikit-Learn website.

Base-Learner	Available Parameters & Ranges	No of Features	Feature Selection Method
**K-Nearest Neighbors (KNN)**	n_neighbors: [2,3,4,5,6,7,8,9,10,15,20,25,30,50,100] weights:[uniform,distance]	f=[5,7,10,12,15,17,20 ,22,25,27,30,40,50,75 ,100,150]	ANOVA F-value
**Perceptron**	max_iter:[5,7,10,12,15,20,25,50,75]	f=[5,7,10,12,15,17,20 ,22,25,27,30,40,50,75 ,100,150]	ANOVA F-value
**Logistic Regression (LR)**	class_weight: [balanced] solver:[sag] max_iter:[5,7,10,12,15,20,25,50,75] C: [1e-5,1e-4,1e-3,1e-2,1e-1,1,1e1,1e2,1e3,1e4,1e5]	f=[5,7,10,12,15,17,20 ,22,25,27,30,40,50,75 ,100,150]	ANOVA F-value
**Elastic Network (EN)**	alpha: [1,0.5,0.1,0.05,0.04,0.03,0.02,0.01,0.005,0.001,0.0001] max_iter:[5,7,10,12,15,20,25,50,75] l1_ratio: [0.01,0.1,0.2,0.3,0.4,0.5,0.6,0.7,0.8,0.9,1] loss:[log] penalty:[elasticnet] class_weight:[balanced] shuffle:[True]	f=[5,7,10,12,15,17,20 ,22,25,27,30,40,50,75 ,100,150]	ANOVA F-value
**Random Forest (RF)**	max_features :np.arange(0.05,0.2,0.001) min_samples_leaf:[2,5,8,10,15,20,25,40,50] n_estimators:[10,20,30,40,50,60,70,80,90,100,125,150,175,200,225,250,275,300,325,350,375,400,425,450,475,500,550,600,650,700] max_depth: [2,4,8,10,None],class_weight:[balanced]	f=[5,7,10,12,15,17,20 ,22,25,27,30,40,50,75 ,100,150]	Random Forest Feature Importance
**Extra Trees (ET)**	max_features :np.arange(0.05,0.2,0.001), min_samples_leaf:[2,5,8,10,15,20,25,40,50], n_estimators:[10,20,30,40,50,60,70,80,90,100,125,150,175,200,225,250,275,300,325,350,375,400,425,450,475,500,550,600,650,700], max_depth: [2,4,8,10,None] class_weight:[balanced]	f=[5,7,10,12,15,17,20 ,22,25,27,30,40,50,75 ,100,150]	Extra Trees Feature Importance
**XGBoost (XGB)**	gamma :[0.01,0.1,1,3,5,7,9,10,15] reg_alpha:[0, 0.001, 0.005, 0.01,0.1,1,3,5] reg_gamma:[0, 0.001, 0.005, 0.01,0.1,1,3,5] learningrate : [0.5,0.1,0.09,0.08,0.07,0.06,0.05,0.04,0.03,0.02,0.01] subsample : [1,0.98,0.95,0.9,0.85,0.8,0.75,0.7] colsample_bytree : [1,0.98,0.95,0.9,0.85,0.8,0.75,0.7] max_depth : [3,4,5,6,7,8,9,10,15] mchildweight :[1,2,3,4,5,6,7,8,9,10,11,12,13,14,15,16,17,18,19,20] n_estimators:[10,20,30,40,50,60,70,80,90,100,125,150,175,200,225,250,275,300,325,350,375,400,425,450,475,500,550,600,650,700]	f=[5,7,10,12,15,17,20 ,22,25,27,30,40,50,75 ,100,150]	XGBoost Feature Importance

Supplementary Table 4. Risk of infection or treatment and event-free survival on Internal Test Cohort. Results for each model were assessed on the internal benchmark (BENCH-I) test cohort – subset of patients in test cohort with full CLL-IPI and a full 2-year (*n*=288) / 5-year (*n*=203) follow-up. CLL-TIM's predictions on BENCH-I were compared to CLL-IPI 2+ and CLL-IPI 4+ where patients with CLL-IPI score of 2 or above and 4 or above were considered as high-risk, respectively. CLL-TIM's High-Confidence (HC) predictions are on a subset of the cohort i.e. on those patients for which CLL-TIM was more confident in (without knowledge of the true outcome). To allow for an equitable comparison, an additional two versions of CLL-IPI score were benchmarked; CLL-IPI with removal of patients in the intermediate-risk category, CLL-IPI NI_4+, and CLL-IPI with removal of the intermediate and high-risk groups, CLL-IPI NIH_7+. This removal had the effect of considering CLL-IPI predictions at the extremes and thus the highest and lowest risk patients according to the CLL-IPI score. CI - Confidence Interval. *includes death as an event

Model (Number of Patients)	Predicted Group	Median event free survival in months (95% CI)*	2-year Infection First Events	2-year Treatment first Events	2-year risk of infection or treatment	2-year event free survival (95% CI)*	2-year Hazard Ratio (95% CI)*
CLL-TIM HC *N*=145	High-Risk	31.375 (23.333 - 50.583)	0.35	0.367	0.717	0.278 (0.184-0.419)	7.269 (7.194 - 7.521)
	Low-Risk	NA (194.75 - NA)	0.082	0.082	0.164	0.835 (0.76-0.918)	
CLL-IPI NI_4+ *N*=168	High-Risk	37.417 (29.75 - 106.583)	0.297	0.297	0.594	0.405 (0.274-0.599)	3.232 (3.049 - 3.756)
	Low-Risk	194.75 (135.917 - NA)	0.142	0.086	0.228	0.765 (0.703-0.834)	
CLL-IPI NIH_7+ *N*=199	High-Risk	12.917 (11.167 - NA)	0.333	0.667	1	0 (0-0)	9.904 (9.863 - 10.154)
	Low-Risk	194.75 (135.917 - NA)	0.142	0.086	0.228	0.765 (0.703-0.834)	
CLL-TIM *N*=288	High-Risk	37.167 (24.083 - 57.667)	0.291	0.337	0.628	0.377 (0.287-0.496)	4.134 (3.972 - 4.495)
	Low-Risk	162 (136 - NA)	0.134	0.069	0.203	0.787 (0.732-0.845)	
CLL-IPI 2+ *N*=288	High-Risk	66.917 (46.833 - 97.333)	0.23	0.23	0.46	0.516 (0.436-0.611)	2.437 (2.163 - 3.052)
	Low-Risk	194.75 (135.917 - NA)	0.142	0.086	0.228	0.765 (0.703-0.834)	
CLL-IPI 4+ *N*=288	High-Risk	37.417 (29.75 - 106.583)	0.297	0.297	0.594	0.429 (0.295-0.624)	2.355 (2.091 - 3.038)
	Low-Risk	124.167 (106.25 - 157.417)	0.163	0.127	0.29	0.7 (0.645-0.759)	

Model (Number of Patients)	Predicted Group	Median event free survival in months (95% CI)*	5-year Infection First Events	5-year Treatment first Events	5-year risk of infection or treatment	5-year event free survival (95% CI)*	5-year Hazard Ratio (95% CI)*
CLL-TIM HC *N*=100	High-Risk	31.375 (23.333 - 50.583)	0.438	0.488	0.926	0.058 (0.017-0.201)	7.869 (7.794 - 8.084)
	Low-Risk	NA (194.75 - NA)	0.182	0.094	0.276	0.709 (0.61-0.825)	
CLL-IPI NI_4+ *N*=110	High-Risk	37.417 (29.75 - 106.583)	0.369	0.359	0.728	0.271 (0.153-0.48)	2.733 (2.502 - 3.313)
	Low-Risk	194.75 (135.917 - NA)	0.285	0.127	0.412	0.561 (0.483-0.652)	
CLL-IPI NIH_7+ *N*=135	High-Risk	12.917 (11.167 - NA)	0.333	0.667	1	0 (0-0)	9.904 (9.863 - 10.154)
	Low-Risk	NA (161.25 - NA)	0.285	0.127	0.412	0.561 (0.483-0.652)	
CLL-TIM *N*=203	High-Risk	37.167 (24.083 - 57.667)	0.378	0.489	0.867	0.091 (0.037-0.226)	3.857 (3.671 - 4.217)
	Low-Risk	162 (136 - NA)	0.285	0.13	0.415	0.546 (0.473-0.63)	
CLL-IPI 2+ *N*=203	High-Risk	66.917 (46.833 - 97.333)	0.349	0.373	0.722	0.226 (0.152-0.336)	2.352 (2.045 - 2.94)
	Low-Risk	194.75 (135.917 - NA)	0.285	0.127	0.412	0.561 (0.483-0.652)	
CLL-IPI 4+ *N*=203	High-Risk	37.417 (29.75 - 106.583)	0.369	0.359	0.728	0.271 (0.153-0.48)	2.015 (1.69 - 2.774)
	Low-Risk	124.167 (106.25 - 157.417)	0.304	0.216	0.52	0.437 (0.371-0.513)	

Supplementary Table 5. Benchmark models description and motivations. Ensembles and prognostic models benchmarked in this work. All ENS- models and CLL-TIM were developed in this work, whereas CLL-IPI 4+/2+/NI_4+/NIH_7+ use scores directly from the CLL-IPI score. With the exception of their unique specifications, all 29 ensembles generated within each protocol, were generated using the same methodology as that used for CLL-TIM (Figure 1). Therefore, the process of base-learner generation, ensemble generation, ensemble scoring and selection of the final ensemble, is ran independently for each protocol. 'Protocol' represents all 29 ensembles generated prior to selection of a final model. 'Selected Ensemble' is the name of top ranked ensemble from each protocol that ranked the highest according to our ensemble ranking score. For instance, ENS-COMP is the protocol that generated 29 ensembles (2-30 base-learners) using the composite outcome as a target outcome, a data-driven approach to feature modelling and patient data prior to CLL diagnosis. CLL-TIM is one of the ensembles generated by ENS-COMP protocol that ranked the highest on our ensemble ranking score prior to opening it on the internal and external test cohorts. 'Target outcome' refers to the outcome the models were originally trained to predict. All models were then analyzed on their ability to predict the composite outcome i.e. outcome of infection or treatment within the next 2-years from the prediction point. The prediction point is defined as 3-months post diagnosis.

**Protocol** (*Selected Ensemble)	**Description & Motivations**	**Target Outcome**	**Feature Modelling**	**Patient Data Look-back from Prediction Point**
**ENS-COMP** (CLL-TIM)	29 Ensemble trained to predict the composite outcome in a data-driven strategy	Composite	Data-Driven	3 months / 1 year / 7 year
**ENS-COMP$_{DoctorsChoice}$** (*ENS-COMP$_{DoctorsChoice}$)	29 Ensembles using a set of feature hand selected by two of our experience CLL physicians. This enables us to compare the validity of a fully-data driven approach like CLL-TIM against that of hand-selected variables. The list of variables available for ENS-COMP$_{DoctorsChoice}$ were: lab test value and date modelling (Supplementary Table 7) of ALC, WBC, neutrophils, platelets, hemoglobin, reticulocytes, lactate dehydrogenase, c-reactive protein, albumin, creatinine, immunoglobulin A, immunoglobulin G, immunoglobulin M, haptoglobin; BOW Modelling (Methods) of Microbiology findings (including blood culture findings); infection data modelling; and Baseline variables: Age, gender, binet stage, β-2 microglobulin, CD38, IGHV status, del(13q), tri(12), del(11q), del(17p), Familial CLL, and ECOG/WHO Performance Status. Therefore, the set of hand-selected variables subsequently underwent the same feature encoding and feature selection processes as those for ENS-COMP and hence CLL-TIM.	Composite	Pre-selected variables only	3 months / 1 year / 7 year
**ENS-COMP$_{3\text{-}months}$** (*ENS-COMP$_{3\text{-}months}$)	29 Ensembles using only data available within the first 3-months of CLL diagnosis. Given that CLL-TIM uses data pre-CLL diagnosis, this enables us to quantify the effect of not having such patient data available.	Composite	Data-Driven	3 months
**ENS-COMP$_{CLLIPI}$** (*ENS-COMP$_{CLLIPI}$)	29 Ensembles using only CLL-IPI variables and thus enabling us to assess the discriminatory power of CLL-IPI variables in the context of an ensemble model trained to predict the composite outcome.	Composite	CLL-IPI variables only	3 months
**ENS-TREAT** (*ENS-Treat)	29 Ensembles trained to predict CLL treatment as an outcome. Enables us to assess whether modelling treatment events is sufficient for predicting infection events prior to CLL treatment	Treatment	Data-Driven	3 months / 1 year / 7 year
**ENS-INFEC** (*ENS-InfecAsFirst)	29 Ensembles trained to predict infection prior to CLL treatment as an outcome. Enables us to assess whether modelling infection as a first event is sufficient for predicting CLL treatment events.	Infection As First	Data-Driven	3 months / 1 year / 7 year
**CLL-IPI 4+**	CLL-IPI Score 4-10 as high risk vs CLL-IPI Score 0-3 as low risk.	Overall Survival (OS) and Time-To-First-Treatment (TTFT)	-	3 months
**CLL-IPI 2+**	CLL-IPI Score 2-10 as high risk vs CLL-IPI Score 0-1 as low risk.	OS/TTFT	-	3 months
**CLL-IPI NI_4+**	CLL-IPI Score 4-10 as high risk vs CLL-IPI Score 0-1 as low risk i.e. removal of CLL-IPI Score 2-3 risk group.	OS/TTFT	-	3 months
**CLL-IPI NIH_7+**	CLL-IPI Score 7-10 as high risk vs CLL-IPI Score 0-1 as low risk i.e. removal of CLL-IPI Score 2-6 risk groups.	OS/TTFT	-	3 months

Supplementary Table 6. 2-Year Composite Outcome for BENCH-I. Predictions were assessed on internal benchmark, BENCH-I – subset of patients in test cohort with full CLL-IPI a full 2-years (n=288). PR-AUC - Area under Precision-Recall Curve, MCC - matthew's correlation coefficient, TP, FP, TN, FN are true-positive, false-positive, true-negative, false-negative predictions for 2-year outcome. CLL-TIM's predictions on BENCH-I were compared against CLL-IPI 2+ and CLL-IPI 4+ where patients with CLL-IPI score of 2 or above and 4 or above are considered as high-risk, respectively. CLL-TIM's high-confidence (HC) predictions are on a subset of the BENCH-I cohort i.e. on those patients for which CLL-TIM was more confident in (without knowledge of the true outcome). To allow for an equitable comparison to CLL-IPI, an additional two versions of CLL-IPI score were benchmarked; CLL-IPI with removal of patients in the intermediate-risk category, CLL-IPI NI_4+, and CLL-IPI with removal of the intermediate and high-risk groups, CLL-IPI NIH_7+. This removal had the effect of considering CLL-IPI predictions at the extremes and thus the highest and lowest risk patients according to the CLL-IPI score. Confidence intervals were generated using predictions on 5000 bootstrapped datasets sampled with replacement from the 2-year BENCH-I test cohorts.

	CLL-TIM HC (n=145)	CLL-IPI NIH_7+ (n=168)	CLL-IPI NI_4+ (n=199)	CLL-TIM (n=288)	CLL-IPI 4+ (n=288)	CLL-IPI 2+ (n=288)
PR-AUC	0.778 (0.691 - 0.859)	0.402 (0.323 - 0.487)	0.516 (0.429 - 0.607)	0.662 (0.582 - 0.741)	0.516 (0.447 - 0.589)	0.516 (0.447 - 0.589)
MCC	0.558 (0.418 - 0.697)	0.321 (0.187 - 0.451)	0.312 (0.163 - 0.458)	0.414 (0.3 - 0.525)	0.216 (0.09 - 0.338)	0.245 (0.131 - 0.353)
TPs	42.969 (37.0 - 49.0)	5.973 (2.0 - 11.0)	21.995 (15.0 - 30.0)	53.937 (44.0 - 63.0)	22.076 (14.0 - 30.0)	57.939 (48.0 - 67.0)
FPs	16.941 (10.0 - 24.0)	0.0 (0.0 - 0.0)	14.993 (8.0 - 22.0)	32.012 (22.0 - 42.0)	15.146 (8.0 - 23.0)	67.971 (55.0 - 81.0)
TNs	71.059 (64.0 - 78.0)	125.0 (125.0 - 125.0)	125.007 (118.0 - 132.0)	160.988 (151.0 - 171.0)	177.854 (170.0 - 185.0)	125.029 (112.0 - 138.0)
FNs	14.031 (8.0 - 20.0)	37.027 (32.0 - 41.0)	37.005 (29.0 - 44.0)	41.063 (32.0 - 51.0)	72.924 (65.0 - 81.0)	37.061 (28.0 - 47.0)
Precision	0.719 (0.631 - 0.815)	1.0 (1.0 - 1.0)	0.596 (0.457 - 0.735)	0.629 (0.545 - 0.718)	0.594 (0.447 - 0.743)	0.461 (0.4 - 0.523)
Recall	0.754 (0.649 - 0.86)	0.139 (0.047 - 0.256)	0.373 (0.254 - 0.508)	0.568 (0.463 - 0.663)	0.232 (0.147 - 0.316)	0.61 (0.505 - 0.705)

Supplementary Table 7. Significance Testing for Model Comparison on 2-Year Composite Outcome for BENCH-I. Comparisons were performed using a one-tailed Mann-Whitney U test on the difference in PR-AUC / MCC over the 5000 bootstrapped datasets. CLL-TIM's high-confidence (HC) predictions are on a subset of the BENCH-I cohort i.e. on those patients for which CLL-TIM was more confident in (without knowledge of the true outcome). To allow for an equitable comparison to CLL-IPI, an additional two versions of CLL-IPI score were benchmarked; CLL-IPI with removal of patients in the intermediate-risk category, CLL-IPI NI_4+, and CLL-IPI with removal of the intermediate and high-risk groups, CLL-IPI NIH_7+. *** p<0.0005, – identical distribution. Results are shown for 'PR-AUC / MCC'.

	CLL-TIM HC (n=145)	CLL-IPI NIH_7+ (n=168)	CLL-IPI NI_4+ (n=199)
CLL-TIM HC (n=145)	- / -	*** / ***	*** / ***
CLL-IPI NIH_7+ (n=168)	/	- / -	/ ***
CLL-IPI NI_4+ (n=199)	/	*** / ***	- / -

Supplementary Table 8. Significance Testing for Model Comparison on 2-Year Composite Outcome for BENCH-I. Comparisons were performed using a one-tailed Wilcoxon signed-rank test on the difference PR-AUC / MCC over the 5000 bootstrapped datasets . CLL-TIM's predictions on BENCH-I were compared against CLL-IPI 2+ and CLL-IPI 4+ where patients with CLL-IPI score of 2 or above and 4 or above are considered as high-risk, respectively. *** p<0.0005, – identical distribution. Results are shown for 'PR-AUC / MCC'.

	CLL-TIM (n=288)	CLL-IPI 4+ (n=288)	CLL-IPI 2+ (n=288)
CLL-TIM (n=288)	- / -	*** / ***	*** / ***
CLL-IPI 4+ (n=288)	/	- / -	- /
CLL-IPI 2+ (n=288)	/	- / ***	- / -

Supplementary Table 9. 5-Year Composite Outcome for BENCH-I. Predictions were assessed on internal benchmark, BENCH-I – subset of patients in test cohort with full CLL-IPI a full 5-years (n=203). PR-AUC - Area under Precision-Recall Curve, MCC - matthew's correlation coefficient, TP, FP, TN, FN are true-positive, false-positive, true-negative, false-negative predictions for 5-year outcome. CLL-TIM's predictions on BENCH-I were compared against CLL-IPI 2+ and CLL-IPI 4+ where patients with CLL-IPI score of 2 or above and 4 or above are considered as high-risk, respectively. CLL-TIM's high-confidence (HC) predictions are on a subset of the BENCH-I cohort i.e. on those patients for which CLL-TIM was more confident in (without knowledge of the true outcome). To allow for an equitable comparison to CLL-IPI, an additional two versions of CLL-IPI score were benchmarked; CLL-IPI with removal of patients in the intermediate-risk category, CLL-IPI NI_4+, and CLL-IPI with removal of the intermediate and high-risk groups, CLL-IPI NIH_7+. This removal had the effect of considering CLL-IPI predictions at the extremes and thus the highest and lowest risk patients according to the CLL-IPI score. Confidence intervals were generated using predictions on 5000 bootstrapped datasets sampled with replacement from the 5-year BENCH-I test cohorts.

	CLL-TIM HC (n=100)	CLL-IPI NIH_7+ (n=110)	CLL-IPI NI_4+ (n=135)	CLL-TIM (n=203)	CLL-IPI 4+ (n=203)	CLL-IPI 2+ (n=203)
PR-AUC	0.935 (0.893 - 0.97)	0.571 (0.433 - 0.7)	51.95 (44.0 - 59.0)	1.987 (0.0 - 5.0)	25.013 (22.0 - 27.0)	21.05 (14.0 - 29.0)
MCC	0.691 (0.642 - 0.745)	0.192 (0.111 - 0.272)	5.988 (2.0 - 11.0)	0.0 (0.0 - 0.0)	44.0 (44.0 - 44.0)	60.012 (55.0 - 64.0)
TPs	0.755 (0.702 - 0.806)	0.23 (0.082 - 0.36)	26.013 (18.0 - 34.0)	4.966 (1.0 - 9.0)	44.034 (40.0 - 48.0)	59.987 (51.0 - 68.0)
FPs	0.89 (0.85 - 0.924)	0.385 (0.283 - 0.475)	69.001 (57.0 - 81.0)	4.999 (1.0 - 10.0)	56.001 (51.0 - 60.0)	72.999 (61.0 - 85.0)
TNs	0.809 (0.761 - 0.854)	0.13 (0.006 - 0.237)	26.113 (17.0 - 35.0)	4.993 (1.0 - 9.0)	56.007 (52.0 - 60.0)	115.887 (107.0 - 125.0)
FNs	0.809 (0.761 - 0.854)	0.275 (0.141 - 0.401)	82.083 (71.0 - 93.0)	17.042 (10.0 - 24.0)	43.958 (37.0 - 51.0)	59.917 (48.0 - 71.0)
Precision	0.935 (0.893 - 0.97)	0.571 (0.433 - 0.7)	51.95 (44.0 - 59.0)	1.987 (0.0 - 5.0)	25.013 (22.0 - 27.0)	21.05 (14.0 - 29.0)
Recall	0.691 (0.642 - 0.745)	0.192 (0.111 - 0.272)	5.988 (2.0 - 11.0)	0.0 (0.0 - 0.0)	44.0 (44.0 - 44.0)	60.012 (55.0 - 64.0)

Supplementary Table 10. Significance Testing for Model Comparison on 5-Year Composite Outcome for BENCH-I. Comparisons were performed using a one-tailed Mann-Whitney U test on the difference in PR-AUC / MCC over the 5000 bootstrapped datasets. CLL-TIM's high-confidence (HC) predictions are on a subset of the BENCH-I cohort i.e. on those patients for which CLL-TIM was more confident in (without knowledge of the true outcome). To allow for an equitable comparison to CLL-IPI, an additional two versions of CLL-IPI score were benchmarked; CLL-IPI with removal of patients in the intermediate-risk category, CLL-IPI NI_4+, and CLL-IPI with removal of the intermediate and high-risk groups, CLL-IPI NIH_7+. *** p<0.0005, – identical distribution. Results are shown for 'PR-AUC / MCC'.

	CLL-TIM HC (n=100)	CLL-IPI NIH_7+ (n=110)	CLL-IPI NI_4+ (n=135)
CLL-TIM HC (n=100)	- / -	*** / ***	*** / ***
CLL-IPI NIH_7+ (n=110)	/	- / -	/
CLL-IPI NI_4+ (n=135)	/	*** / ***	- / -

Supplementary Table 11. Significance Testing for Model Comparison on 5-Year Composite Outcome for BENCH-I. Comparisons were performed using a one-tailed Wilcoxon signed-rank test on the difference PR-AUC / MCC over the 5000 bootstrapped datasets . CLL-TIM's predictions on BENCH-I were compared against CLL-IPI 2+ and CLL-IPI 4+ where patients with CLL-IPI score of 2 or above and 4 or above are considered as high-risk, respectively. *** p<0.0005, – identical distribution. Results are shown for 'PR-AUC / MCC'.

	CLL-TIM (n=203)	CLL-IPI 4+ (n=203)	CLL-IPI 2+ (n=203)
CLL-TIM (n=203)	- / -	*** / ***	*** / ***
CLL-IPI 4+ (n=203)	/	- / -	- /
CLL-IPI 2+ (n=203)	/	- / ***	- / -

Supplementary Table 12. Risk of infection or treatment and event-free survival on External Cohort. Results for each model were assessed on the external benchmark (BENCH-E) - subset of patients in external German CLL7 cohort with full CLL-IPI and a full 2-year (*n*=281) / 5-year (*n*=182) follow-up. CLL-TIM's predictions on BENCH-E were compared to CLL-IPI 2+ and CLLIPI 4+ where patients with CLL-IPI score of 2 or above and 4 or above are considered as high-risk, respectively. CLL-TIMT20-30's predictions are on a subset of the cohort i.e. top 20% ranked as high-risk and top 30% ranked as low-risk). To allow for an equitable comparison, an additional two versions of CLL-IPI score were benchmarked; CLL-IPI with removal of patients in the intermediate risk category, CLL-IPI NI_4+, and CLL-IPI with removal of the intermediate and high-risk groups, CLL-IPI NIH_7+. This removal had the effect of considering CLL-IPI predictions at the extremes and thus the highest and lowest risk patients according to the CLL-IPI score. CLL-IPI NIH_7+ was not included in this analysis as no predictions on BENCH-E had a CLL-IPI score of 7 or above. CI - Confidence Interval. *Includes death as an event.

Model (Number of Patients)	Predicted Group	Median event free survival in months (95% CI)	2-year Infection First Events	2-year Treatment first Events	2-year risk of infection or treatment	2-year event free survival (95% CI)	2-year Hazard Ratio (95% CI)
CLL-TIM $_{T20-30}$ *N*=142	High-Risk	41.167 (31.917 - 67.5)	0.158	0.386	0.544	0.561 (0.412 - 0.673)	2.851 (2.649 - 3.46)
	Low-Risk	161.333 (101.75 - NA)	0.176	0.047	0.223	0.247 (0.15 - 0.333)	
CLL-IPI NI_4+ *N*=229	High-Risk	21.083 (7.833 - NA)	0.375	0.375	0.75	0.75 (0.17 - 0.925)	5.541 (5.464 - 5.964)
	Low-Risk	167.417 (147.75 - NA)	0.175	0.066	0.241	0.25 (0.189 - 0.306)	
CLL-TIM *N*=281	High-Risk	22 (16.75 - NA)	0.125	0.75	0.875	0.875 (0.218 - 0.98)	5.522 (5.439 - 5.917)
	Low-Risk	149.083 (109.333 - 173.833)	0.165	0.11	0.275	0.286 (0.23 - 0.337)	
CLL-IPI 4+ *N*=281	High-Risk	21.083 (7.833 - NA)	0.375	0.375	0.75	0.75 (0.17 - 0.925)	4.646 (4.553 - 5.142)
	Low-Risk	149.083 (109.333 - 173.833)	0.158	0.121	0.279	0.289 (0.233 - 0.341)	
CLL-TIM 3+Matched *N*=281	High-Risk	34.083 (16.75 - 79.25)	0.111	0.444	0.555	0.556 (0.322 - 0.708)	2.829 (2.627 - 3.447)
	Low-Risk	161 (121.583 - NA)	0.169	0.094	0.263	0.276 (0.219 - 0.329)	
CLL-IPI 3+ *N*=281	High-Risk	67.5 (40.417 - 94.917)	0.13	0.319	0.449	0.464 (0.332 - 0.569)	2.21 (1.918 - 2.912)
	Low-Risk	167.417 (147.75 - NA)	0.175	0.066	0.241	0.25 (0.189 - 0.306)	

Model (Number of Patients)	Predicted Group	Median event free survival in months (95% CI)	5-year Infection First Events	5-year Treatment first Events	5-year risk of infection or treatment	5-year event free survival (95% CI)	5-year Hazard Ratio (95% CI)
CLL-TIM $_{T20-30}$ *N*=107	High-Risk	41.167 (31.917 - 67.5)	0.218	0.559	0.777	0.812 (0.668 - 0.894)	3.05 (2.841 - 3.563)
	Low-Risk	161.333 (101.75 - NA)	0.337	0.092	0.429	0.453 (0.327 - 0.556)	
CLL-IPI NI_4+ *N*=139	High-Risk	21.083 (7.833 - NA)	0.375	0.625	1	1 (NA - NA)	7.3 (7.235 - 7.589)
	Low-Risk	167.417 (147.75 - NA)	0.341	0.097	0.438	0.448 (0.367 - 0.518)	
CLL-TIM *N*=182	High-Risk	22 (16.75 - NA)	0.125	0.875	1	1 (NA - NA)	5.503 (5.415 - 5.879)
	Low-Risk	149.083 (109.333 - 173.833)	0.322	0.169	0.491	0.505 (0.434 - 0.568)	
CLL-IPI 4+ *N*=182	High-Risk	21.083 (7.833 - NA)	0.375	0.625	1	1 (NA - NA)	6.081 (6.001 - 6.422)
	Low-Risk	149.083 (109.333 - 173.833)	0.315	0.177	0.492	0.506 (0.434 - 0.569)	
CLL-TIM 3+Matched *N*=182	High-Risk	34.083 (16.75 - 79.25)	0.157	0.713	0.87	0.907 (0.674 - 0.974)	3.623 (3.446 - 4.054)
	Low-Risk	161 (121.583 - NA)	0.334	0.133	0.467	0.479 (0.404 - 0.544)	
CLL-IPI 3+ *N*=182	High-Risk	67.5 (40.417 - 94.917)	0.242	0.476	0.718	0.747 (0.597 - 0.841)	2.405 (2.114 - 2.999)
	Low-Risk	167.417 (147.75 - NA)	0.341	0.097	0.438	0.448 (0.367 - 0.518)	

Supplementary Table 13. 2-Year Composite Outcome for BENCH-E. Predictions were assessed on external benchmark, BENCH-E – subset of patients in external test cohort with full CLL-IPI a full 2-years (n=281). PR-AUC - Area under Precision-Recall Curve, MCC - matthew's correlation coefficient, TP, FP, TN, FN are true-positive, false-positive, true-negative, false-negative predictions for 2-year outcome. CLL-TIM's predictions on BENCH-E were compared against CLL-IPI 4+ where patients with CLL-IPI score of 4 or above are considered as high-risk, respectively. For CLL-TIM 3+Matched, we re-calibrated CLL-TIM's threshold for high-risk predictions until the number of high-risk predictions equaled those achieved by CLL-IPI 3+. This enables comparison of both methods when the same amount of high-risk predictions are put forward. CLL-TIM $T_{20\text{-}30}$'s predictions are on a subset of the cohort i.e. i.e. top 20% ranked as high-risk and top 30% ranked as low-risk). To allow for an equitable comparison to CLL-IPI, an additional two versions of CLL-IPI score were benchmarked; CLL-IPI with removal of patients in the intermediate-risk category, CLL-IPI NI_4+, and CLL-IPI with removal of the intermediate and high-risk groups, CLL-IPI NIH_7+. This removal had the effect of considering CLL-IPI predictions at the extremes and thus the highest and lowest risk patients according to the CLL-IPI score. Confidence intervals were generated using predictions on 5000 bootstrapped datasets sampled with replacement from the 2-year BENCH-E test cohorts.

	CLL-TIM $T_{20\text{-}30}$ (n=142)	CLL-IPI NIH_7+ (n=221)	CLL-IPI NI_4+ (n=229)	CLL-TIM (n=281)	CLL-IPI 4+ (n=281)	CLL-TIM 3+Matched (n=281)	CLL-IPI 3+ (n=281)
PR-AUC	0.572 (0.448 - 0.697)	0.258 (0.21 - 0.313)	0.343 (0.269 - 0.431)	0.483 (0.392 - 0.578)	0.405 (0.338 - 0.486)	0.483 (0.392 - 0.578)	0.405 (0.338 - 0.486)
MCC	0.335 (0.175 - 0.491)	0.0 (0.0 - 0.0)	0.216 (0.055 - 0.353)	0.219 (0.091 - 0.321)	0.171 (0.032 - 0.285)	0.191 (0.057 - 0.322)	0.165 (0.037 - 0.295)
TPs	31.144 (23.0 - 40.0)	0.0 (0.0 - 0.0)	6.018 (2.0 - 11.0)	7.084 (3.0 - 12.0)	6.018 (2.0 - 11.0)	15.108 (9.0 - 22.0)	14.091 (8.0 - 21.0)
FPs	25.963 (17.0 - 35.0)	0.0 (0.0 - 0.0)	1.974 (0.0 - 5.0)	0.998 (0.0 - 3.0)	1.974 (0.0 - 5.0)	12.005 (6.0 - 19.0)	12.923 (7.0 - 20.0)
TNs	66.974 (54.0 - 80.0)	161.004 (150.0 - 171.0)	161.004 (150.0 - 171.0)	198.002 (196.0 - 199.0)	197.026 (194.0 - 199.0)	186.995 (180.0 - 193.0)	186.077 (179.0 - 192.0)
FNs	18.856 (12.0 - 27.0)	50.939 (42.0 - 59.0)	50.939 (42.0 - 59.0)	74.916 (70.0 - 79.0)	75.982 (71.0 - 80.0)	66.892 (60.0 - 73.0)	67.909 (61.0 - 74.0)
Precision	0.546 (0.435 - 0.66)	0 (0 - 0)	0.753 (0.4 - 1.0)	0.878 (0.6 - 1.0)	0.753 (0.4 - 1.0)	0.558 (0.381 - 0.741)	0.523 (0.346 - 0.708)
Recall	0.623 (0.481 - 0.756)	0.0 (0.0 - 0.0)	0.106 (0.034 - 0.192)	0.086 (0.037 - 0.146)	0.073 (0.024 - 0.134)	0.184 (0.11 - 0.268)	0.172 (0.098 - 0.256)

Supplementary Table 14. Significance Testing for Model Comparison on 2-Year Composite Outcome for BENCH-E. Comparisons were performed using a one-tailed Mann-Whitney U test on the difference in PR-AUC / MCC over the 5000 bootstrapped datasets. CLL-TIM $T_{20\text{-}30}$'s predictions are on a subset of the cohort i.e. i.e. top 20% ranked as high-risk and top 30% ranked as low-risk). To allow for an equitable comparison to CLL-IPI, an additional two versions of CLL-IPI score were benchmarked; CLL-IPI with removal of patients in the intermediate-risk category, CLL-IPI NI_4+, and CLL-IPI with removal of the intermediate and high-risk groups, CLL-IPI NIH_7+. This removal had the effect of considering CLL-IPI predictions at the extremes and thus th32e highest and lowest risk patients according to the CLL-IPI score. *** p<0.0005, – identical distribution. Results are shown for 'PR-AUC / MCC'.

	CLL-TIM $T_{20\text{-}30}$ (n=142)	CLL-IPI NIH_7+ (n=221)	CLL-IPI NI_4+ (n=229)
CLL-TIM $T_{20\text{-}30}$ (n=142)	- / -	***/ ***	***/ ***
CLL-IPI NIH_7+ (n=221)	/	- / -	/
CLL-IPI NI_4+ (n=229)	/	***/ ***	- / -

Supplementary Table 15. Significance Testing for Model Comparison on 2-Year Composite Outcome for BENCH-E. Comparisons were performed using a one-tailed Wilcoxon signed-rank test on the difference PR-AUC / MCC over the 5000 bootstrapped datasets . . CLL-TIM's predictions on BENCH-E were compared against CLL-IPI 4+ where patients with CLL-IPI score of 4 or above are considered as high-risk, respectively. For CLL-TIM 3+Matched, we re-calibrated CLL-TIM's threshold for high-risk predictions until the number of high-risk predictions equaled those achieved by CLL-IPI 3+. This enables comparison of both methods when the same amount of high-risk predictions are put forward. *** p<0.0005, – identical distribution. Results are shown for 'PR-AUC / MCC'.

	CLL-TIM (n=281)	CLL-IPI 4+ (n=281)		CLL-TIM 3+Matched (*n*=281)	CLL-IPI 3+ (*n*=281)
CLL-TIM (n=281)	- / -	*** / ***	CLL-TIM 3+Matched (n=281)	- / -	*** / ***
CLL-IPI 4+ (n=281)	/	- / -	CLL-IPI 3+ (n=281)	/	- / -

Supplementary Table 16. 5-Year Composite Outcome for BENCH-E. Predictions were assessed on external benchmark, BENCH-E – subset of patients in external test cohort with full CLL-IPI a full 5-years (n=182). PR-AUC - Area under Precision-Recall Curve, MCC - matthew's correlation coefficient, TP, FP, TN, FN are true-positive, false-positive, true-negative, false-negative predictions for 5-year outcome. CLL-TIM's predictions on BENCH-E were compared against CLL-IPI 4+ where patients with CLL-IPI score of 4 or above are considered as high-risk, respectively. For CLL-TIM 3+Matched, we re-calibrated CLL-TIM's threshold for high-risk predictions until the number of high-risk predictions equaled those achieved by CLL-IPI 3+. This enables comparison of both methods when the same amount of high-risk predictions are put forward. CLL-TIM $T_{20\text{-}30}$'s predictions are on a subset of the cohort i.e. top 20% ranked as high-risk and top 30% ranked as low-risk. The thresholds for these were derived on the 2-year outcome, and same patients were followed up until the 5-year outcome. To allow for an equitable comparison to CLL-IPI, an additional two versions of CLL-IPI score were benchmarked; CLL-IPI with removal of patients in the intermediate-risk category, CLL-IPI NI_4+, and CLL-IPI with removal of the intermediate and high-risk groups, CLL-IPI NIH_7+. This removal had the effect of considering CLL-IPI predictions at the extremes and thus the highest and lowest risk patients according to the CLL-IPI score. Confidence intervals were generated using predictions on 5000 bootstrapped datasets sampled with replacement from the 5-year BENCH-E test cohorts.

	CLL-TIM $T_{20\text{-}30}$ (n=107)	CLL-IPI NIH_7+ (n=131)	CLL-IPI NI_4+ (n=139)	CLL-TIM (n=182)	CLL-IPI 4+ (n=182)	CLL-TIM 3+Matched (n=182)	CLL-IPI 3+ (n=182)
PR-AUC	0.863 (0.797 - 0.92)	0.667 (0.614 - 0.723)	0.723 (0.674 - 0.771)	0.843 (0.795 - 0.889)	0.809 (0.769 - 0.848)	0.843 (0.795 - 0.889)	0.809 (0.769 - 0.848)
MCC	0.355 (0.193 - 0.504)	0.0 (0.0 - 0.0)	0.175 (0.107 - 0.237)	0.133 (0.082 - 0.183)	0.134 (0.082 - 0.183)	0.196 (0.11 - 0.266)	0.159 (0.047 - 0.246)
TPs	42.937 (33.0 - 54.0)	0.0 (0.0 - 0.0)	8.051 (3.0 - 14.0)	7.922 (3.0 - 14.0)	8.051 (3.0 - 14.0)	20.955 (13.0 - 30.0)	19.957 (12.0 - 28.0)
FPs	4.978 (1.0 - 9.0)	0.0 (0.0 - 0.0)	0.0 (0.0 - 0.0)	0.0 (0.0 - 0.0)	0.0 (0.0 - 0.0)	1.001 (0.0 - 3.0)	2.003 (0.0 - 5.0)
TNs	25.0 (18.0 - 32.0)	47.022 (43.0 - 51.0)	47.022 (43.0 - 51.0)	52.0 (52.0 - 52.0)	52.0 (52.0 - 52.0)	50.999 (49.0 - 52.0)	49.997 (47.0 - 52.0)
FNs	33.937 (25.0 - 44.0)	84.118 (74.0 - 95.0)	84.118 (74.0 - 95.0)	122.078 (116.0 - 127.0)	121.949 (116.0 - 127.0)	109.045 (100.0 - 117.0)	110.043 (102.0 - 118.0)
Precision	0.897 (0.81 - 0.977)	0.0 (0.0 - 0.0)	1 (1.0 - 1.0)	1 (1.0 - 1.0)	1 (1.0 - 1.0)	0.955 (0.857 - 1.0)	0.909 (0.778 - 1.0)
Recall	0.558 (0.447 - 0.667)	0.0 (0.0 - 0.0)	0.087 (0.033 - 0.149)	0.061 (0.023 - 0.108)	0.062 (0.023 - 0.108)	0.161 (0.1 - 0.231)	0.154 (0.092 - 0.215)

Supplementary Table 17. Significance Testing for Model Comparison on 5-Year Composite Outcome for BENCH-E. Comparisons were performed using a one-tailed Mann-Whitney U test on the difference in PR-AUC / MCC over the 5000 bootstrapped datasets. CLL-TIM $T_{20\text{-}30}$'s predictions are on a subset of the cohort i.e. top 20% ranked as high-risk and top 30% ranked as low-risk. The thresholds for these were derived on the 2-year outcome, and same patients were followed up until the 5-year outcome To allow for an equitable comparison to CLL-IPI, an additional two versions of CLL-IPI score were benchmarked; CLL-IPI with removal of patients in the intermediate-risk category, CLL-IPI NI_4+, and CLL-IPI with removal of the intermediate and high-risk groups, CLL-IPI NIH_7+. This removal had the effect of considering CLL-IPI predictions at the extremes and thus the highest and lowest risk patients according to the CLL-IPI score. *** p<0.0005, – identical distribution. Results are shown for 'PR-AUC / MCC'.

	CLL-TIM $T_{20\text{-}30}$ (n=107)	CLL-IPI NIH_7+ (n=131)	CLL-IPI NI_4+ (n=139)
CLL-TIM $T_{20\text{-}30}$ (n=107)	- / -	***/ ***	***/ ***
CLL-IPI NIH_7+ (n=131)	/	- / -	/
CLL-IPI NI_4+ (n=139)	/	***/ ***	- / -

Supplementary Table 18. Significance Testing for Model Comparison on 5-Year Composite Outcome for BENCH-E. Comparisons were performed using a one-tailed Wilcoxon signed-rank test on the difference PR-AUC / MCC over the 5000 bootstrapped datasets . CLL-TIM's predictions on BENCH-E were compared against CLL-IPI 4+ where patients with CLL-IPI score of 4 or above are considered as high-risk, respectively. For CLL-TIM 3+Matched, we re-calibrated CLL-TIM's threshold for high-risk predictions until the number of high-risk predictions equaled those achieved by CLL-IPI 3+. This enables comparison of both methods when the same amount of high-risk predictions are put forward. *** p<0.0005, – identical distribution. Results are shown for 'PR-AUC / MCC'.

	CLL-TIM (n=182)	CLL-IPI 4+ (n=182)		CLL-TIM 3+Matched (n=182)	CLL-IPI 3+ (n=182)
CLL-TIM (n=182)	- / -	*** /	CLL-TIM 3+Matched (n=182)	- / -	*** / ***
CLL-IPI 4+ (n=182)	/ ***	- / -	CLL-IPI 3+ (n=182)	/	- / -

Supplementary Table 19. 2-Year Treatment Outcome for BENCH-I. Predictions were assessed on internal benchmark, BENCH-I – subset of patients in test cohort with full CLL-IPI a full 2-years (n=288). PR-AUC - Area under Precision-Recall Curve, MCC - matthew's correlation coefficient, TP, FP, TN, FN are true-positive, false-positive, true-negative, false-negative predictions for 2-year outcome. CLL-TIM's predictions on BENCH-I were compared against CLL-IPI 2+ and CLL-IPI 4+ where patients with CLL-IPI score of 2 or above and 4 or above are considered as high-risk, respectively. CLL-TIM's high-confidence (HC) predictions are on a subset of the BENCH-I cohort i.e. on those patients for which CLL-TIM was more confident in (without knowledge of the true outcome). To allow for an equitable comparison to CLL-IPI, an additional two versions of CLL-IPI score were benchmarked; CLL-IPI with removal of patients in the intermediate-risk category, CLL-IPI NI_4+, and CLL-IPI with removal of the intermediate and high-risk groups, CLL-IPI NIH_7+. This removal had the effect of considering CLL-IPI predictions at the extremes and thus the highest and lowest risk patients according to the CLL-IPI score. Confidence intervals were generated using predictions on 5000 bootstrapped datasets sampled with replacement from the 2-year BENCH-I test cohorts.

	CLL-TIM HC (n=145)	CLL-IPI NIH_7+ (n=168)	CLL-IPI NI_4+ (n=199)	CLL-TIM (n=288)	CLL-IPI 4+ (n=288)	CLL-IPI 2+ (n=288)
PR-AUC	0.51 (0.371 - 0.648)	0.257 (0.142 - 0.42)	0.359 (0.233 - 0.517)	0.425 (0.316 - 0.538)	0.338 (0.247 - 0.447)	0.338 (0.247 - 0.447)
MCC	0.381 (0.229 - 0.512)	0.323 (0.063 - 0.529)	0.303 (0.136 - 0.469)	0.315 (0.194 - 0.434)	0.203 (0.061 - 0.347)	0.232 (0.125 - 0.339)
TPs	23.937 (19.0 - 28.0)	4.023 (1.0 - 8.0)	13.974 (9.0 - 19.0)	31.059 (24.0 - 38.0)	14.069 (8.0 - 20.0)	34.943 (28.0 - 41.0)
FPs	36.091 (26.0 - 46.0)	2.004 (0.0 - 5.0)	23.087 (15.0 - 32.0)	55.055 (43.0 - 68.0)	23.135 (15.0 - 33.0)	91.12 (76.0 - 106.0)
TNs	77.909 (68.0 - 88.0)	145.996 (143.0 - 148.0)	145.913 (137.0 - 154.0)	181.945 (169.0 - 194.0)	213.865 (204.0 - 222.0)	145.88 (131.0 - 161.0)
FNs	7.063 (3.0 - 12.0)	15.977 (12.0 - 19.0)	16.026 (11.0 - 21.0)	19.941 (13.0 - 27.0)	36.931 (30.0 - 43.0)	16.057 (10.0 - 23.0)
Precision	0.401 (0.323 - 0.482)	0.667 (0.25 - 1.0)	0.379 (0.255 - 0.514)	0.362 (0.289 - 0.438)	0.38 (0.238 - 0.528)	0.278 (0.23 - 0.328)
Recall	0.772 (0.613 - 0.903)	0.201 (0.05 - 0.4)	0.466 (0.3 - 0.633)	0.609 (0.471 - 0.745)	0.276 (0.157 - 0.392)	0.685 (0.549 - 0.804)

Supplementary Table 20. Significance Testing for Model Comparison on 2-Year Treatment Outcome for BENCH-I. Comparisons were performed using a one-tailed Mann-Whitney U test on the difference in PR-AUC / MCC over the 5000 bootstrapped datasets. CLL-TIM's high-confidence (HC) predictions are on a subset of the BENCH-I cohort i.e. on those patients for which CLL-TIM was more confident in (without knowledge of the true outcome). To allow for an equitable comparison to CLL-IPI, an additional two versions of CLL-IPI score were benchmarked; CLL-IPI with removal of patients in the intermediate-risk category, CLL-IPI NI_4+, and CLL-IPI with removal of the intermediate and high-risk groups, CLL-IPI NIH_7+. *** p<0.0005, – identical distribution. Results are shown for 'PR-AUC / MCC'.

	CLL-TIM HC (n=145)	CLL-IPI NIH_7+ (n=168)	CLL-IPI NI_4+ (n=199)
CLL-TIM HC (n=145)	- / -	*** / ***	*** / ***
CLL-IPI NIH_7+ (n=168)	/	- / -	/ ***
CLL-IPI NI_4+ (n=199)	/	*** /	- / -

Supplementary Table 21. Significance Testing for Model Comparison on 2-Year Treatment Outcome for BENCH-I. Comparisons were performed using a one-tailed Wilcoxon signed-rank test on the difference PR-AUC / MCC over the 5000 bootstrapped datasets . CLL-TIM's predictions on BENCH-I were compared against CLL-IPI 2+ and CLL-IPI 4+ where patients with CLL-IPI score of 2 or above and 4 or above are considered as high-risk, respectively. *** p<0.0005, – identical distribution. Results are shown for 'PR-AUC / MCC'.

	CLL-TIM (n=288)	CLL-IPI 4+ (n=288)	CLL-IPI 2+ (n=288)
CLL-TIM HC (n=288)	- / -	*** / ***	*** / ***
CLL-IPI 4+ (n=288)	/	- / -	- / ***
CLL-IPI 2+ (n=288)	/	- / ***	- / -

Supplementary Table 22. 2-Year Treatment Outcome for BENCH-E. Predictions were assessed on external benchmark, BENCH-E – subset of patients in external test cohort with full CLL-IPI a full 2-years (n=182). PR-AUC - Area under Precision-Recall Curve, MCC - matthew's correlation coefficient, TP, FP, TN, FN are true-positive, false-positive, true-negative, false-negative predictions for 2-year outcome. CLL-TIM's predictions on BENCH-E were compared against CLL-IPI 4+ where patients with CLL-IPI score of 4 or above are considered as high-risk, respectively. For CLL-TIM 3+Matched, we re-calibrated CLL-TIM's threshold for high-risk predictions until the number of high-risk predictions equaled those achieved by CLL-IPI 3+. This enables comparison of both methods when the same amount of high-risk predictions are put forward. CLL-TIM $T_{20\text{-}30}$'s predictions are on a subset of the cohort i.e. top 20% ranked as high-risk and top 30% ranked as low-risk. To allow for an equitable comparison to CLL-IPI, an additional two versions of CLL-IPI score were benchmarked; CLL-IPI with removal of patients in the intermediate-risk category, CLL-IPI NI_4+, and CLL-IPI with removal of the intermediate and high-risk groups, CLL-IPI NIH_7+. This removal had the effect of considering CLL-IPI predictions at the extremes and thus the highest and lowest risk patients according to the CLL-IPI score. Confidence intervals were generated using predictions on 5000 bootstrapped datasets sampled with replacement from the 2-year BENCH-E test cohorts.

	CLL-TIM $T_{20\text{-}30}$ (n=142)	CLL-IPI NIH_7+ (n=221)	CLL-IPI NI_4+ (n=229)	CLL-TIM (n=281)	CLL-IPI 4+ (n=281)	CLL-TIM 3+Matched (n=281)	CLL-IPI 3+ (n=281)
PR-AUC	0.526 (0.354 - 0.702)	0.073 (0.041 - 0.115)	0.173 (0.068 - 0.342)	0.444 (0.3 - 0.595)	0.29 (0.2 - 0.401)	0.444 (0.3 - 0.595)	0.29 (0.2 - 0.401)
MCC	0.431 (0.284 - 0.56)	0.0 (0.0 - 0.0)	0.213 (-0.039 - 0.448)	0.316 (0.126 - 0.475)	0.124 (-0.052 - 0.293)	0.309 (0.142 - 0.477)	0.237 (0.08 - 0.392)
TPs	22.006 (16.0 - 28.0)	0.0 (0.0 - 0.0)	2.988 (0.0 - 7.0)	6.007 (2.0 - 11.0)	2.988 (0.0 - 7.0)	12.018 (7.0 - 18.0)	10.012 (5.0 - 15.0)
FPs	35.042 (25.0 - 46.0)	0.0 (0.0 - 0.0)	5.004 (1.0 - 10.0)	1.969 (0.0 - 5.0)	5.004 (1.0 - 10.0)	15.033 (8.0 - 23.0)	17.0 (10.0 - 25.0)
TNs	81.918 (68.0 - 97.0)	197.972 (185.0 - 210.0)	197.972 (185.0 - 210.0)	243.031 (240.0 - 245.0)	239.996 (235.0 - 244.0)	229.967 (222.0 - 237.0)	228.0 (220.0 - 235.0)
FNs	4.006 (1.0 - 8.0)	14.029 (8.0 - 20.0)	14.029 (8.0 - 20.0)	29.993 (25.0 - 34.0)	33.012 (29.0 - 36.0)	23.982 (18.0 - 29.0)	25.988 (21.0 - 31.0)
Precision	0.388 (0.29 - 0.49)	0 (0 - 0)	0.374 (0.0 - 0.75)	0.753 (0.417 - 1.0)	0.374 (0.0 - 0.75)	0.447 (0.273 - 0.63)	0.373 (0.208 - 0.545)
Recall	0.846 (0.692 - 0.964)	0.0 (0.0 - 0.0)	0.175 (0.0 - 0.375)	0.167 (0.056 - 0.306)	0.083 (0.0 - 0.194)	0.334 (0.194 - 0.5)	0.278 (0.139 - 0.417)

Supplementary Table 23. Significance Testing for Model Comparison on 2-Year Treatment Outcome for BENCH-E. Comparisons were performed using a one-tailed Mann-Whitney U test on the difference in PR-AUC / MCC over the 5000 bootstrapped datasets. CLL-TIM $T_{20\text{-}30}$'s predictions are on a subset of the cohort i.e. top 20% ranked as high-risk and top 30% ranked as low-risk. To allow for an equitable comparison to CLL-IPI, an additional two versions of CLL-IPI score were benchmarked; CLL-IPI with removal of patients in the intermediate-risk category, CLL-IPI NI_4+, and CLL-IPI with removal of the intermediate and high-risk groups, CLL-IPI NIH_7+. This removal had the effect of considering CLL-IPI predictions at the extremes and thus the highest and lowest risk patients according to the CLL-IPI score. *** p<0.0005, – identical distribution. Results are shown for 'PR-AUC / MCC'.

	CLL-TIM $T_{20\text{-}30}$ (n=142)	CLL-IPI NIH_7+ (n=221)	CLL-IPI NI_4+ (n=229)
CLL-TIM $T_{20\text{-}30}$ (n=142)	- / -	***/ ***	***/ ***
CLL-IPI NIH_7+ (n=221)	/	- / -	/
CLL-IPI NI_4+ (n=229)	/	***/ ***	- / -

Supplementary Table 24. Significance Testing for Model Comparison on 2-Year Treatment Outcome for BENCH-E. Comparisons were performed using a one-tailed Wilcoxon signed-rank test on the difference PR-AUC / MCC over the 5000 bootstrapped datasets . CLL-TIM's predictions on BENCH-E were compared against CLL-IPI 4+ where patients with CLL-IPI score of 4 or above are considered as high-risk, respectively. For CLL-TIM 3+Matched, we re-calibrated CLL-TIM's threshold for high-risk predictions until the number of high-risk predictions equaled those achieved by CLL-IPI 3+. This enables comparison of both methods when the same amount of high-risk predictions are put forward. . *** p<0.0005, – identical distribution. Results are shown for 'PR-AUC / MCC'.

	CLL-TIM (n=281)	CLL-IPI 4+ (n=281)		CLL-TIM 3+Matched (n=281)	CLL-IPI 3+ (n=281)
CLL-TIM (n=281)	- / -	*** / ***	CLL-TIM 3+Matched (n=281)	- / -	*** / ***
CLL-IPI 4+ (n=281)	/	- / -	CLL-IPI 3+ (n=281)	/	- / -

Supplementary Table 25 .Baseline Variables used in this work and expansion using One-Hot-Encoding. All baseline variables taken at time of CLL diagnosis. IGHV - Immunoglobulin heavy-chain variable region. ECOG - Eastern Cooperative Oncology Group. FAMCLL – Familial CLL. Data was sourced from the Danish CLL Registry

Variable Name	One-Hot-Encoding
**Age**	None
**Binet Stage**	A, B, C, NA
**Gender**	Female, Male
**IGHV mutational status**	Mutated, unmutated, NA
**Del(13q)**	Pos, Neg, NA
**Tri(12)**	Pos, Neg, NA
**Del(11q)**	Pos, Neg, NA
**Del(17p)**	Pos, Neg, NA
**ECOG Performance Status**	0, 1, 2, 3, 4, NA
**FAMCLL**	Pos, Neg, NA
**β-2 microglobulin >4.0 mg/L**	Pos, Neg, NA
**ZAP70>20%**	Pos, Neg, NA
**CD38>30%**	Pos, Neg, NA

Supplementary Table 26. Ensemble Ranking Score. Describes the six criteria that were used to score each ensemble generated by the genetic algorithm. The highest ranked ensemble according to this score was chosen as the final model with which the test cohort was then opened. Both the internal training and validation cohorts were used as part of the score. The rank of each ensemble was calculated as the average score over the six criteria after standardization of each. * To account for multiple comparisons, the Benjamini-Hochberg correction was applied with a False Discovery Rate (FDR) of 1%[1].

Criteria	Sub-criteria	Description
**Composition dissimilarity of base-learners**	a. Number of unique target outcomes in the ensemble.	In this work, base-learners were trained on 3 different target outcomes: The composite outcome of infection or CLL treatment, infection as first outcome, and CLL treatment outcome. For each unique outcome included in the ensemble, a score of one point is given or added.
	b. Number of unique algorithms in the ensemble.	Seven classes of machine learning classifiers were trained as base-learners. For each unique algorithm included in the ensemble, a score of one point is given or added.
**Predictive dissimilarity of base-learners**	a. Base-learner to base-learner predictive dissimilarity on training cohort	Differences in classifier performance on the training cohort were assessed using paired *T*-test over the mean Matthew's correlation coefficient (MCC) over 5 repetitions of 10-Fold cross-validation (CV) according to Bouckaert and colleagues[2] – 5 repetitions instead of 10 were used for computational efficiency. The average *number* of dissimilar base-learners with $p<0.05^*$ was then extracted as the final score.
	b. Base-learner to Base-learner predictive dissimilarity on validation Cohort.	Dissimilarity in base-learner predictions on the validation cohort was performed with McNemar's Test[3]. The average *number* of dissimilar base-learners with $p<0.05^*$ was then extracted as the final score.
	c. Ensemble to base-learner predictive dissimilarity on validation cohort.	Dissimilarity in base-learner to ensemble predictions on the validation cohort was performed with McNemar's Test. The average *number* of dissimilar base-learners with $p<0.05^*$ was then extracted as the final score.
**Average Feature Size of Base-Learners**	None	Given that base-learner feature set sizes range from 7 to 150, the average feature size of the ensemble is taken as the average feature size of all base-learners in the ensemble.
**Base-learner Performance on Validation Cohort.**	None	MCC of the ensemble on the validation cohort.
**Base-learner Performance on Training Cohort.**	None	Average MCC of the results of 5 x 10-Fold cross-validation (CV). Given that hyper-parameters of the base-learners were not tuned to the cross-validation folds, and feature selection was employed within each fold, this renders CV results viable for estimating performance.
**Ensemble Size (No. of Base−learners)**	None	A score ranging from 2-30 depending on the ensemble size. As long as independence between the base-learners is up kept, ensembles of increasing further reduce generalization error[439]. Therefore, ensemble size was also included as a criterion.

Supplementary Discussion

Role of Immunoglobulins and Bag-of-Words Features

None of CLL-TIM's base-learners made use of immunoglobulins and 'Doctor's Choice' models that included immunoglobulins, were outperformed (Supplementary Fig. 7). Immunoglobulins were also substantially outranked by many other features in our univariate analyses (Supplementary Data 3-4). These results are in contrast to previous work on immunoglobulins[5–8]. It is possible, that their importance was previously overestimated as they were only benchmarked against a handful of variables. BOW features that modelled pathology and diagnosis were selected by our data-driven strategy but had small effects on performance (Supplementary Figs. 8 and 12). Patients with a high number of rare pathologies showed only a minor increase in risk of infection and treatment (Supplementary Fig. 16). It cannot be ascertained to which extent this is due to comorbidity or other events modelled by this feature. Access to larger patient cohorts in the future may enable pathology and diagnosis information to be modelled with word embeddings that may better exploit relationships between these types of categorical features[55].

Supplementary Methods

Feature Generation

To model missing values, we encoded baseline variables using one-hot-encoding. For the rest of the four data sources, we encoded patient data into features that summarize patient events within three look-back windows of 3 months, 1 year, and the entire patient's history spanning a maximum of 7 years (Fig. 1a). Bag-Of-Words (BOW) modeling[9] was applied to the microbiology, blood culture, pathology and diagnosis datasets (Fig. 2c). For any given patient, this BOW vector details the number of times that an event (or word) has happened within each look-back window. The vocabulary of words for the most common findings in the CLL training cohort (Supplementary Fig. 1) includes 9 words for blood culture findings, 46 words for microbiology findings (that exclude blood cultures), 216 diagnosis coding words and 153 words for pathology codes. For each dataset, we kept only common words present in more than 1% of the CLL population in the training cohort. For each of the data sources, the remaining long tail of uncommon words were assigned to a rare category as a proxy to comorbidity. This rare category included 1,170 diagnoses, 1,419 pathology codes, 3 microbiology findings and 1 blood culture finding. Based on medical review of the highest ranked uncommon words in the rare categories for pathology and diagnoses, we hypothesized that these could be used as a proxy for comorbidity. Even though the actual number of rare words may not directly represent the rate of comorbidity (given the additional noise of non-comorbidity related words and different words pointing to the same finding), patients with a high rare word count were hypothesized to be more likely to have a higher number of

comorbidity related events than those with a low rare word count. Using the BOW paradigm, each patient was thus represented using a vector with the length of all the combined vocabularies (i.e. 424 words). To avoid future-time leakage, we removed from the 'Pathology' and 'Diagnosis' data sets, words indicating infection, surgical procedures, bone marrow biopsy or transplantation. These included pathology SNOMED codes P28260, P28270, P28280, T06000, F06006, M35330, M00020 and P31002 as well as diagnosis codes AZCL3, DC911 and AZCK0. Due to differences in nomenclature, distinct words pointing to identical findings were in fact left untouched, as we strived for a fully data-driven approach without interference. Any words with high correlation were however removed as part of the data-cleaning process (Supplementary Table 2). We designed several features that captured the density, recentness and distribution of previous infections (Fig. 2c and Supplementary Fig. 3). Irrespective of the finding, infection was defined as having a blood culture drawn[5]. Laboratory tests in the were reduced to 183 common laboratory tests by restricting only those tests that were available for more than 1% of the CLL population in the training cohort. For each laboratory test, we encoded changes over time using least-squares-fitting curves that encoded linear and exponential-like changes over time[10], along with minimum, maximum and average values (Supplementary Table 1). Skewness and kurtosis were also used to describe changes over time which deviate from a symmetric normal distriubtion[11]. We also modeled information that can be held in the act of taking a laboratory test. Effectively, the physician's decision to take a test may be either routine, or a latent variable capturing the urgency of a patient's condition and acute symptoms as interpreted by the physician. For instance, a recent prostate-specific antigen (PSA) test may be indicative of symptoms of prostate cancer, the subsequent initiation of anitbiotics[12], or even anxiety and stress as a result of being advised to take such a test[13]. The frequency of a given laboratory test has been shown to correlate with mortality and several diagnoses in the MIMIC-III datasets[14] and in another study, an increased number of tests taken with very low-variance in the last year held information related to urgency of a situation[15]. Therefore, we encoded the dates of laboratory tests into features (number of tests taken, recentness and spread of testing dates), irrespective of the result of the laboratory test (Supplementary Table 1). The potentiality of these variables may be better understood in the context of a multivariable model combining several features together. In total, the feature encoding process described above resulted in a set of 640 extra variables that were encoded into 7,288 features as summarized in Supplementary Table 2. The 7,288 features encoded from our five data sources were not all part of the final CLL-TIM ensemble, rather the 7,288 features form the initial set of features available for modelling prior to feature selection strategies described in 'Base-learner Generation'.

Supplementary References

1. Benjamini, Y. & Hochberg, Y. Controlling the false discovery rate: A practical and powerful approach to multiple testing. *Journal of the Royal Statistical Society: Series B (Methodological)* **57,** 289–300 (1995).
2. Bouckaert, R. R. & Frank, E. in *Advances in knowledge discovery and data mining* (eds. Dai, H., Srikant, R. & Zhang, C.) **3056,** 3–12 (Springer Berlin Heidelberg, 2004).
3. McNemar, Q. Note on the sampling error of the difference between correlated proportions or percentages. *Psychometrika* **12,** 153–157 (1947).
4. Hansen, L. K. & Salamon, P. Neural network ensembles. *IEEE Trans. Pattern Anal. Mach. Intell.* **12,** 993–1001 (1990).
5. Andersen, M. A. *et al.* Incidence and predictors of infection among patients prior to treatment of chronic lymphocytic leukemia: a Danish nationwide cohort study. *Haematologica* **103,** e300–e303 (2018).
6. Crassini, K. R. *et al.* Humoral immune failure defined by immunoglobulin class and immunoglobulin G subclass deficiency is associated with shorter treatment-free and overall survival in Chronic Lymphocytic Leukaemia. *Br. J. Haematol.* **181,** 97–101 (2018).
7. Crassini, K. R., Best, O. G. & Mulligan, S. P. Immune failure, infection and survival in chronic lymphocytic leukemia. *Haematologica* **103,** e329 (2018).
8. Ishdorj, G. *et al.* IgA levels at diagnosis predict for infections, time to treatment, and survival in chronic lymphocytic leukemia. *Blood Adv.* **3,** 2188–2198 (2019).
9. Sivic & Zisserman. Video Google: a text retrieval approach to object matching in videos. in *Proceedings Ninth IEEE International Conference on Computer Vision* 1470–1477 vol.2 (IEEE, 2003). doi:10.1109/ICCV.2003.1238663
10. Gordon, S. P. & Yang, Y. Approximating exponential and logarithmic functions using polynomial interpolation. *Int. J. Math. Educ. Sci. Technol.* **48,** 455–473 (2017).
11. Lambrou, T., Kudumakis, P., Speller, R., Sandler, M. & Linney, A. Classification of audio signals using statistical features on time and wavelet transform domains. in *Proceedings of the 1998 IEEE International Conference on Acoustics, Speech and Signal Processing, ICASSP' ' '98 (Cat. No.98CH36181)* 3621–3624 (IEEE, 1998). doi:10.1109/ICASSP.1998.679665
12. Scardino, P. T. The responsible use of antibiotics for an elevated PSA level. *Nat. Rev. Urol.* **4,** 1–1 (2007).
13. Kotwal, A. A., Schumm, P., Mohile, S. G. & Dale, W. The influence of stress, depression, and anxiety on PSA screening rates in a nationally representative sample. *Med. Care* **50,** 1037–1044 (2012).
14. Che, Z., Purushotham, S., Cho, K., Sontag, D. & Liu, Y. Recurrent Neural Networks for Multivariate Time Series with Missing Values. *Sci. Rep.* **8,** 6085 (2018).
15. Loekito, E. *et al.* Common laboratory tests predict imminent medical emergency team calls, intensive care unit admission or death in emergency department patients. *Emerg Med Australas* **25,** 132–139 (2013).
