# Supplementary material for: Machine learning can identify newly diagnosed patients with CLL at high risk of infection
Source: Nat Commun. 2020 Jan 17;11:363. doi: 10.1038/s41467-019-14225-8 (PMC6969150; doi:10.1038/s41467-019-14225-8)
Supplement: Supplementary file 3 — Reporting Summary [file 41467_2019_14225_MOESM3_ESM.pdf]

nature research

Corresponding author(s): Carsten Utoft Niemann

Last updated by author(s): Dec 1, 2019

Reporting Summary

Nature Research wishes to improve the reproducibility of the work that we publish. This form provides structure for consistency and transparency in reporting. For further information on Nature Research policies, seeAuthors & Referees and theEditorial Policy Checklist .

Statistics

For all statistical analyses, confirm that the following items are present in the figure legend, table legend, main text, or Methods section.

n/a	Confirmed	
☐	☒	The exact sample size (*n*) for each experimental group/condition, given as a discrete number and unit of measurement
☒	☐	A statement on whether measurements were taken from distinct samples or whether the same sample was measured repeatedly
☐	☒	The statistical test(s) used AND whether they are one- or two-sided<br>*Only common tests should be described solely by name; describe more complex techniques in the Methods section.*
☐	☒	A description of all covariates tested
☐	☒	A description of any assumptions or corrections, such as tests of normality and adjustment for multiple comparisons
☐	☒	A full description of the statistical parameters including central tendency (e.g. means) or other basic estimates (e.g. regression coefficient) AND variation (e.g. standard deviation) or associated estimates of uncertainty (e.g. confidence intervals)
☐	☒	For null hypothesis testing, the test statistic (e.g. *F*, *t*, *r*) with confidence intervals, effect sizes, degrees of freedom and *P* value noted<br>*Give P values as exact values whenever suitable.*
☒	☐	For Bayesian analysis, information on the choice of priors and Markov chain Monte Carlo settings
☒	☐	For hierarchical and complex designs, identification of the appropriate level for tests and full reporting of outcomes
☐	☒	Estimates of effect sizes (e.g. Cohen's *d*, Pearson's *r*), indicating how they were calculated

Our web collection on statistics for biologists contains articles on many of the points above.

Software and code

Policy information about availability of computer code

Data collection	Data was collected from the Danish National Registry an PERSIMUNE warehouse (http://www.persimune.dk/)
Data analysis	Code for CLL-TIM feature encoding, base-learner prediction and generation of personalized risk-factors is available at https://github.com/RA19/clltim. An online version is available at CLL-TIM.org.

For manuscripts utilizing custom algorithms or software that are central to the research but not yet described in published literature, software must be made available to editors/reviewers. We strongly encourage code deposition in a community repository (e.g. GitHub). See the Nature Research guidelines for submitting code & software for further information.

Data

Policy information about availability of data

All manuscripts must include a data availability statement. This statement should provide the following information, where applicable:

- Accession codes, unique identifiers, or web links for publicly available datasets
- A list of figures that have associated raw data
- A description of any restrictions on data availability

The authors declare that the individual patient level data that support the findings of this study are available from the corresponding author upon reasonable request. As the individual patient level data cannot be anonymized, only pseudonymized, according to Danish and EU legislation, the data cannot be deposited in a public repository. However, the authors provide a data repository with individual patient level data that can be made available with 2-factor authentication for researchers on request.

Field-specific reporting

Please select the one below that is the best fit for your research. If you are not sure, read the appropriate sections before making your selection.

[x] Life sciences   [ ] Behavioural & social sciences   [ ] Ecological, evolutionary & environmental sciences

For a reference copy of the document with all sections, see nature.com/documents/nr-reporting-summary-flat.pdf

Life sciences study design

All studies must disclose on these points even when the disclosure is negative.

Sample size	Sample size was determined Based on the Danish National CLL registry, we identified 4,149 patients diagnosed with CLL between January 2004 and July 2017.
Data exclusions	To ensure inclusion of all results from tests ordered at time of diagnosis we shifted time-point zero, which we refer to as the prediction point, to three months post-diagnosis for the training phase. We thus excluded patients who died (n=74) or initiated CLL treatment (n=373) prior to this (Extended Data Fig.1), reducing the available sample size to n=3,720 (see Table 1 for baseline characteristics)
Replication	We provide code for CLL-TIM https://github.com/RA19/clltim and web-application for anyone to get CLL-TIM predictions at CLL-TIM.org. We test CLL-TIM's generalizability on blind test internal and external cohorts
Randomization	Using stratified sampling that preserved class distributions, we randomly divided the cohort into a training set of 2,432 (65%) patients and equally sized internal validation and test sets (approx. 17.5% each at 642 and 646 patients, respectively). For the external cohort, all patients available were predicted on.
Blinding	Blinding is not relevant to the study

Reporting for specific materials, systems and methods

We require information from authors about some types of materials, experimental systems and methods used in many studies. Here, indicate whether each material, system or method listed is relevant to your study. If you are not sure if a list item applies to your research, read the appropriate section before selecting a response.

Materials & experimental systems

n/a	Involved in the study	
[x]	[ ]	Antibodies
[x]	[ ]	Eukaryotic cell lines
[x]	[ ]	Palaeontology
[x]	[ ]	Animals and other organisms
[x]	[ ]	Human research participants
[x]	[ ]	Clinical data

Methods

n/a	Involved in the study	
[x]	[ ]	ChIP-seq
[x]	[ ]	Flow cytometry
[x]	[ ]	MRI-based neuroimaging
