# Supplementary material for: Machine learning can identify newly diagnosed patients with CLL at high risk of infection
Source: Nat Commun. 2020 Jan 17;11:363. doi: 10.1038/s41467-019-14225-8 (PMC6969150; doi:10.1038/s41467-019-14225-8)
Supplement: Supplementary file 4 — Description of Additional Supplementary Files [file 41467_2019_14225_MOESM4_ESM.pdf]

Description of Additional Supplementary Files

File Name: Supplementary Data 1
Description: **Ranked CLL-TIM features using shapely additive explanations (SHAP).** For any given feature, the SHAP value (averaged across 28 baselearners) quantifies the contribution of that feature towards predicting a patient as high or low risk of a composite event within 2 years from 3 months post CLL diagnosis using CLL-TIM. 'Contribution to Composite Outcome' are the absolute SHAP values averaged across all patients in the internal training, validation and test cohort. 'Contribution to Infection Prior to CLL Treatment (A)' are the absolute SHAP values averaged across patients with infection prior to treatment that CLL-TIM predicted as highrisk. 'Contribution to CLL Treatment Prior to Infection (B)' are the absolute SHAP values averaged across patients with CLL treatment prior to infection that CLL-TIM predicted as high-risk. (A)-(B) represents the specific contribution towards infection events (positive values) and the specific contribution towards treatment events (negative values). 'Infection Specific' are the top risk factors that are specific to infection. 'Treatment Specific' are the top risk factors that are specific to treatment. These subsets of features satisfied two conditions i) (A)-(B) was significant (one-tailed p0.005. The threshold of 0.005 was chosen on the basis that the top 50 contributors to the composite outcome have values >0.005.

File Name: Supplementary Data 2
Description: **Univariate analysis on CLL-TIM's 228 features for the composite outcome.** We performed univariate analysis on the 228 features that are part of CLL-TIM using four methods of Kruskal-Wallis H-Test, Mann-Whitney UTest, ANOVA F-Test and Student's T-Test. Shown are the top 10 variables ranked according to p-value. Excluded were features for which the number of high-risk instances (patients) was lower than 100. Testing was performed on the all 3720 Danish CLL patients (i.e. training, validation and test cohorts) post CLL-TIM analysis and none of this information was used to build CLL-TIM. We performed no accounting for multiple-testing as we were only interested in the relative p-values. No normality tests were performed before application of any statistical test, instead we used nonparametric tests of Kruskal-Wallis H-Test and Mann-Whitney U-Test. All tests were two-tailed.

File Name: Supplementary Data 3
Description: **Univariate analysis on 7288 features for the composite outcome.** We performed univariate analysis on the 7288 features generated in this work using four methods of Kruskal-Wallis H-Test, Mann-Whitney U-Test, ANOVA F-Test and Student's T-Test. Shown are the top 10 variables ranked according to pvalue. Also shown are the top-ranked features for Immunoglobulin A (IgA), IgG and IgM. Excluded were features for which the number of high-risk instances (patients) was lower than 100. Testing was performed on the all 3720 Danish CLL patients (i.e. training, validation and test cohorts) post CLL-TIM analysis and none of this information was used to build CLL-TIM. We performed no accounting for multiple-testing as we were only interested in the relative p-values. No normality tests were performed before application of any statistical test, instead we used non-parametric tests of Kruskal-Wallis H-Test and Mann-Whitney U-Test. All tests were two-tailed.

File Name: Supplementary Data 4
Description: **Univariate analysis on 7288 features for the infection outcome.** We performed univariate analysis on the 7288 features generated in this work using four methods of Kruskal-Wallis H-Test, Mann-Whitney U-Test, ANOVA F-Test and Student's T-Test. Shown are the top 10 variables

ranked according to pvalue. Also shown are the top-ranked features for Immunoglobulin A (IgA), IgG and IgM. Excluded were features for which the number of high-risk instances (patients) was lower than 100. Testing was performed on the all 3720 Danish CLL patients (i.e. training, validation and test cohorts) post CLL-TIM analysis and none of this information was used to build CLL-TIM. We performed no accounting for multiple-testing as we were only interested in the relative p-values. No normality tests were performed before application of any statistical test, instead we used non-parametric tests of Kruskal-Wallis H-Test and Mann-Whitney U-Test. All tests were two-tailed.
